# Machine Learning Classification Models for the Design of Novel Nitroimidazole Derivatives with Anti-*Trichomonas vaginalis* Activity

**DOI:** 10.1021/acsomega.6c05649

**Published:** 2026-07-23

**Authors:** Gabriel Corrêa Veríssimo, Anand Miranda Antoniassi, Laura Gonçalves Rezende, Victor Rocha Lamego, Wanderleya Toledo dos Santos, Ana Cláudia Chagas de Paula, Lauren Hubert Jaeger, Thales Kronenberger, Renata Barbosa de Oliveira, Vinícius Gonçalves Maltarollo

**Affiliations:** † Departamento de Produtos Farmacêuticos, Faculdade de Farmácia, Universidade Federal de Minas Gerais, Av. Antõnio Carlos 6627, Pampulha, Belo Horizonte, Minas Gerais 31270-901, Brazil; ‡ Postgraduate Programme in Bioinformatics, Federal University of Minas Gerais (UFMG), Belo Horizonte 31270-901, Brazil; § Programa de Pós-graduação Em Ciências Farmacêuticas, Faculdade de Farmácia, 28113 Universidade Federal de Juiz de Fora, Rua José Lourenço Kelmer, S/N, Campus Universitário, São Pedro, Juiz de Fora, Minas Gerais 36036-900, Brazil; ∥ Laboratório de Células-Tronco E Parasitologia, Faculdade de Farmácia, Universidade Federal de Juiz de Fora, Rua José Lourenço Kelmer, S/N, Campus Universitário, São Pedro, Juiz de Fora, Minas Gerais 36036-900, Brazil; ⊥ Institute of Medical Microbiology and Hygiene, Interfaculty Institute of Microbiology and Infection Medicine, 27203 Eberhard Karls Universitat Tübingen, Tübingen 72076, Germany; # German Center for Infection Research (DZIF), Partner-Site Tübingen, Elfriede-Aulhorn-Str. 6, Tübingen 72076, Germany; ∇ School of Pharmacy, Faculty of Health Sciences, University of Eastern Finland, Kuopio 70211, Finland

## Abstract

*Trichomonas vaginalis* is the pathogen responsible for trichomoniasis, a widespread sexually transmitted infection worldwide. Treatment currently relies solely on nitroimidazole-class drugs, such as metronidazole and tinidazole, to which resistance is increasingly reported. When resistance or treatment failure occurs, higher doses are prescribed, which in turn heightens the risk of toxic side effects. Therefore, discovering new therapeutic options is urgently needed. To address this, quantitative structure–activity relationship (QSAR) models were developed using nitroimidazole derivatives to analyze the relationship between various chemical structures and their efficacy against the parasite, selectivity between two different strains, and potential cytotoxicity against HeLa cells. Three distinct models were selected, demonstrating strong performance in both internal and external validation (external MCC values ranging from 0.583 to 0.849). These classification models were then applied to predict the biological activity of 57 nitroimidazole compounds, aiming to identify promising new candidates for treating *T. vaginalis* infections. Three selected hit compounds were synthesized and experimentally validated against *T. vaginalis* as well as HeLa cells. All compounds were active, with IC_50_ values ranging from 0.17 to 48.92 μM, with no cytotoxicity for HeLa cells observed at the tested concentrations, showing them to be promising candidates for preclinical evaluation.

## Graphical abstract

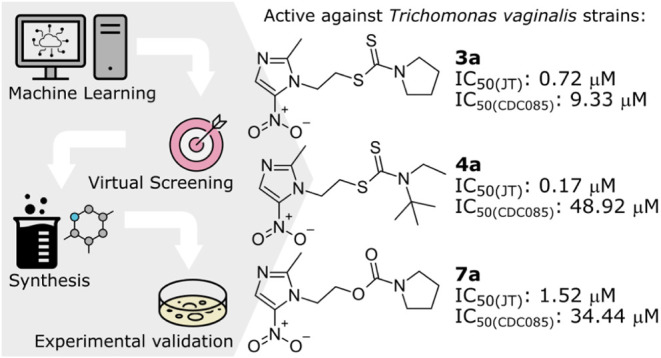

## 1 Introduction

*Trichomonas vaginalis* is a parasitic protozoan that inhabits the human urogenital tract and causes an infection known as trichomoniasis. Trichomoniasis is the most widespread nonviral sexually transmitted infection, with World Health Organization (WHO) estimations rounding to about 156 million new cases annually,^1^ thereby posing this condition as a significant global health concern.^2^ Trichomoniasis can appear in multiple clinical forms, including inflammation of the vagina (vaginitis), cervix (cervicitis), vulva (vulvitis), urethra (urethritis), and prostate (prostatitis), as well as symptoms like itching (pruritus). In addition, *T. vaginalis* infection has been linked to increased HIV susceptibility, a higher likelihood of cervical cancer, and infertility, with the highest burden registered in marginalized populations.^3^ These health complications can result in physical discomfort and contribute to emotional and social difficulties that diminish overall quality of life.^4,5^

Current trichomoniasis treatment relies exclusively on pharmacotherapy, and the nitroimidazole-based compounds are the only drugs authorized by the US Food and Drug Administration (FDA). Agents such as metronidazole (MTZ), tinidazole, and secnidazole belong to this class, which are effective and inexpensive, being associated with mild adverse effects only when used in high dosages.^6,7^ Their antimicrobial action is broad, extending beyond *T. vaginalis* to include other anaerobic protozoa such as *Entamoeba histolytica*, *Giardia duodenalis*, and *Blastocystis hominis*, as well as anaerobic bacteria such as *Bacteroides* spp., *Clostridium* spp., and *Helicobacter pylori*.^8^

Metronidazole acts as a prodrug that requires enzymatic reduction to become effective. This activation occurs in the hydrogenosome, an organelle involved in anaerobic energy production. Within this organelle, pyruvate is decarboxylated by pyruvate:ferredoxin oxidoreductase, yielding acetate and enabling ATP synthesis. During this reaction, electrons are transferred to ferredoxin (Fd), which is subsequently reoxidized by hydrogenase (Hyd) to produce molecular hydrogen. Metronidazole and related compounds interfere by accepting electrons from Fd, resulting in the generation of harmful nitro radicals. Another metabolic route involves the malate enzyme, which is NAD^+^, and also participates in metronidazole reduction within the hydrogenosome. The precise cellular targets of these toxic radicals in *T. vaginalis* are not fully characterized, though it is believed they primarily attack nucleic acids, proteins, and other essential molecules.^9,10^

Nitroimidazole resistance in *T. vaginalis* occurs through two primary mechanisms: aerobic and anaerobic. The aerobic resistance mechanism is most significant clinically and involves diminished ferredoxin function and/or disruption in oxygen-reducing systems. This results in increased oxygen levels within the hydrogenosome, which inhibits the reduction process of the drug’s nitro group.^11,12^ The anaerobic resistance mechanism involves the suppression or complete loss of pyruvate ferredoxin reductase and/or hydrogenase activity, often due to gene deletion in resistant strains. However, this form of resistance is considered less impactful since metronidazole activation can be performed by other enzymes. Resistance to treatment was first reported in 1962, and the number of resistant strains has since increased, with current data showing that at least 5% of trichomoniasis cases involve drug-resistant *T. vaginalis*.^13^ In such instances, higher doses are typically administered, though this may increase the risk of side effects. The rise in resistance underscores the urgent need for new drugs and therapeutic strategies. Although numerous nitroimidazole derivatives have been synthesized with promising efficacy, none have yet demonstrated sufficient clinical benefit to replace metronidazole and tinidazole as the standard treatments. Additionally, toxicity and tolerability concerns remain important challenges, particularly at higher doses or with prolonged treatment regimens, further highlighting the need for safer therapeutic alternatives.^14,15^

Because of the growing resistance and the limitations of currently available therapies, it becomes essential to explore new approaches that can accelerate the identification of more effective compounds. In this context, innovative strategies have gained prominence, particularly those that enable a more rational and efficient optimization of the drug discovery process. The process of drug discovery has undergone significant changes over time and now benefits from the support of computational methods. Among these tools, the classical QSAR, alone or in combination with machine learning (ML), remains relevant, especially when interfacing with phenotypical screening or predicting new drugs in a target-independent fashion.^16–18^

Our previous QSAR models, developed to predict the activity of metronidazole derivatives, showed that bulkier substituent groups near the piperidine ring are unfavorable.^19^ Likewise, contour maps generated from comparative molecular field analysis (CoMFA) suggested similar steric and electrostatic influences on ligand binding,^20^ with negative contributions to the activity, particularly from long alkyl chains positioned away from the core metronidazole ring.

Our previous approach^19^ combined Hologram Quantitative Structure–Activity Relationship (HQSAR) and Random Forest-based QSAR techniques to understand structural features responsible for the *T. vaginalis* activity. Our models predicted three nitroimidazole analogs with potential activity against resistant strains. In the present study, we aimed to (i) generate a library of derivative compounds from those reported in Veríssimo’s work^19^ focusing on synthetic feasibility (Figure 1); (ii) train, validate, and interpret ML-based QSAR models to predict *T. vaginalis* and cytotoxicity activities; (iii) use some selected models for screening potential hit compounds; (iv) synthesize and characterize those selected hits; and (v) finally, validate them experimentally against two *T. vaginalis* strains, as well as assess the cytotoxicity of the candidate compounds.

**Figure 1:**
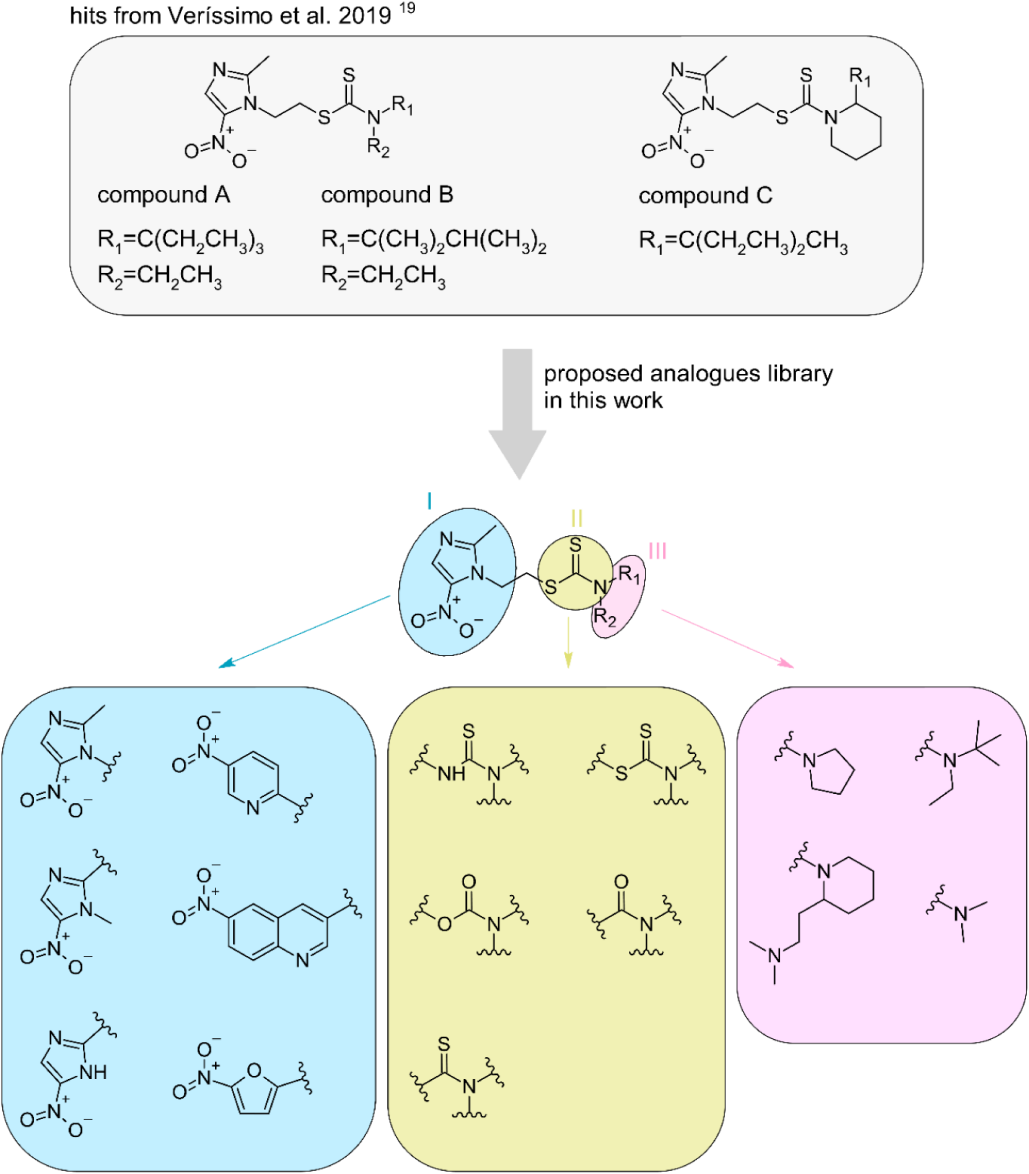
Three original hits (compounds A, B, and C) from Veríssimo’s previous work^19^ and proposed molecular modifications based on synthetic feasibility and availability of reactants used to propose the molecular library in this current work. The analogues library was generated by combining modifications in the nitroimidazole ring (teal, region I), the dithiocarbamate (yellow, region II), and its substituents (pink, region III).

## 2 Materials and Methods

### 2.1 Machine Learning-Based QSAR Modeling

#### 2.1.1 Dataset and Fingerprint Calculations

105 compounds were retrieved from the literature, aiming to construct three distinct datasets for training ML classification models.^20–22^ The datasets generated were: (i) the sensitivity data set, aimed at developing models to evaluate the activity of molecules against *T. vaginalis* sensitive strains; (ii) the resistance dataset, designed to generate models capable of assessing the activity of nitroimidazole derivatives against *T. vaginalis* strains resistant to metronidazole; and (iii) the toxicity dataset, intended to evaluate the cytotoxicity of nitroimidazoles in HeLa cells. The biological activities were binarized using a cutoff of 50 μM for both *T. vaginalis* datasets and 5,000 μM for the HeLa cells toxicity dataset in order to build classification models.

Further, 3D chemical structures were generated and prepared by calculating the lowest energy conformer and the most probable protonation state at pH 7.4 using OMEGA 2.5.1.4 ^23,24^ and QUACPAC 1.6.3.1,^25^ respectively. Afterward, PaDEL-Descriptor software^26^ was then used to calculate the following molecular fingerprints: AtomPairs2D, AtomPairs2D count, EState, CDK Fingerprint, CDK ExtendedFingerprint, CDK GraphOnly Fingerprint, Klekota-Roth, Klekota-Roth count, MACCS, PubChem, Substructure, and Substructure count.

#### 2.1.2 Splitting into Training and Test Sets

The datasets were divided into training and test subsets in an 80:20 ratio, based on a modified version of the MASSA Algorithm v.0.9.6.^27^ This procedure ensures that both subsets are representative of the entire dataset in terms of chemical diversity, biological response, and key physicochemical properties. Overall, the division was guided by hierarchical cluster analysis (HCA) across three dimensions: (i) drug-likeness properties, including molecular weight, predicted logarithm of n-octanol–water partition coefficient (logP), number of hydrogen bond donors and acceptors, rotatable bonds, sp^3^ carbon fraction, and topological polar surface area; (ii) structural diversity, assessed via PubChem-based fingerprints; and (iii) biological activity distribution. This approach guarantees that both training and test sets retain the diversity and characteristics of the original dataset.

#### 2.1.3 Class Balancing Strategy

The proportion of active/inactive data in all three datasets was unbalanced; therefore, the SMOTE (synthetic minority oversampling technique)^28,29^ method, implemented on the KNIME platform,^30^ was employed to balance the datasets.

#### 2.1.4 Machine Learning Models

The ML models were generated using a workflow implemented on the KNIME platform.^30^ For each dataset, classification models were developed using a combination of distinct machine learning algorithms, their intrinsic hyperparameter variations, and calculated fingerprint sets as descriptors: decision tree (DT), random forest (RF), multilayer perceptron (MLP), and k-nearest neighbors (kNN). For comparative purposes, all generated models were evaluated both with and without data balancing by using the SMOTE technique. The variation of hyperparameters was based on previously reported works^31,32^ and is listed below:

**Decision Tree –** The minimum number of records per internal node varied, ranging from 1 to 15.

**Random Forest**: maximum depth and the total number of trees varied within the range of 10 to 50 levels and 10,000 to 50,000 trees, respectively.

**Multilayer Perceptron –** The number of hidden layers and the number of hidden neurons per layer varied from 1 to 5 hidden layers and from 5 to 20 hidden neurons per layer.

**k-Nearest Neighbors**: The number of neighbors to consider (*k*), varying this parameter within the range of 1 to 27, using only odd numbers.

#### 2.1.5 Performance of Models

We conducted both internal (5-fold cross-validation) and external validations to assess the performance of the developed models. The evaluation was carried out using the following metrics: Matthews Correlation Coefficient (MCC, eq 1, ranging from −1 to 1), F1-Score (eq 2), the area under the curve (AUC) of a receiver operating characteristic curve (ROC), true positive rate (TPR), and true negative rate (TNR) (eqs 3 and 4, respectively).^33^

1 MCC=TP×TN−FP×FN/sqrt(TP+FP)(TP+FN)(TN+FP)(TN+FN)

2 F1‐score=2TP/2TP−FP−FN

3 TPR=TP/(TP+FN)

4 TNR=TN/(TN+FP)

where TP = true positives, TN = true negatives, FP = false positives, and FN = false negatives.

The resulting models were primarily ranked according to the average value of internal and external MCC, with secondary criteria being the mean internal and external F1-Score and the mean internal and external AUC ROC values, based on previously reported strategies.^32,34,35^

#### 2.1.6 Model’s Interpretation

The selected models were interpreted using the variable exchange method:^36^ (i) for each variable (fingerprint bin), 5 models were generated and externally validated, keeping all data original but randomizing that variable; (ii) then, the difference between the extMCC of the original selected model and the average MCC value for models generated with that randomized variable was calculated; (iii) variables with an MCC difference higher than zero proceeded to the next step; (iv) median values of that variable for both classes (active and inactive) were calculated; (v) variables with median values higher for active compounds were defined as contributing positively to the model, and vice versa; (vi) finally, a last filter to select only variables with significant contributions was applied by calculating and retaining only fingerprint bins with p-values lower than 0.05 (calculated using the Mann–Whitney method^37^). Lastly, the RDKit node for identifying SMARTS patterns and highlighting training set compounds was used for coloring substructures according to the contribution of those variables to the model prediction.

#### 2.1.7 Applicability Domain

The applicability domain of most predictive models was assessed by principal component analysis using the X-data (fingerprint set) employed for training those models through the KNIME implementation. Then, the chemical space of the training set compounds and the designed compounds was visually compared using a lower-dimensional (2D) data projection, as described in the literature.^38,39^

### 2.2 Virtual Screening

After the steps of data balancing, training, validation, and interpretation, the most predictive models were selected for each dataset. These models were used to predict the activity of the 57 nitroimidazole derivatives and to select hit compounds for synthesis and *in vitro* assays.

#### 2.2.1 Design and Preparation of New Nitroimidazole Derivatives

Based on three unconfirmed hits from our previous study,^19^ a set of 57 new analogues was proposed, taking into account the availability of reagents and synthetic routes accessible within our laboratory. After the generation of the new analogues in the SMILES format, the most energetically stable 3D structures were generated, and protonation states at pH 7.4 were calculated using OMEGA 2.5.1.4 ^23,24^ and QUACPAC 1.6.3.1,^25^ respectively.

### 2.3 Quantum Chemistry Calculations

The *ab initio* quantum mechanical calculations were carried out for the highest occupied molecular orbital (HOMO) and lowest unoccupied molecular orbital (LUMO) energy calculations for hit compounds and control compounds reported in Veríssimo et al.^19^ using density functional theory (DFT) with the B3LYP functional^40^ and the 6-311+G­(d,p) basis set,^41^ as implemented in Jaguar (version 2024.3, Schrödinger, LLC, New York, NY, 2017, version 4).^42^ Initial conformations of representative ligands were subjected to geometry optimization via energy minimization in an aqueous environment using an implicit PBF solvation model. Subsequently, molecular orbital energies were calculated, and their corresponding plots were generated.

### 2.4 Chemistry

Commercial sources provided the analytical reagents employed in the synthesis method; no further purification procedure was used. Analytical thin-layer chromatography (TLC) was carried out on 0.5 mm plates that had been previously coated with a suspension formed by 8 g of silica gel 60 with 13% w/w CaSO_4_ (Sigma-Aldrich) in 19 mL of distilled water. The melting points (mp) were determined on a Microquímica MQAPF301 apparatus. The infrared (IR) spectra were recorded using a Spectrum One PerkinElmer FT-IR spectrometer equipped with an ATR system. The NMR spectra were recorded on a Bruker AVANCE III HD NanoBay or OneBay (400 MHz) spectrometer and measured at a temperature of 300.15 K (27 °C). Tetramethylsilane (TMS) was used as an internal standard. Chemical shifts are expressed on the δ (ppm) scale, and *J* values are given in Hz, with the multiplicity of signals referred to as singlet (s), doublet (d), triplet (t), quartet (q), double doublet (dd), and multiplet (m). Compounds **1a,** **2a**,^43^ **3a**,^22^ **5a,** and **6a** ^44^ have been previously described in the literature. Their syntheses were carried out following the reported methodologies, with slight modifications where necessary.

#### 2.4.1 Synthesis of N-Tert-butyl-N-ethyl­{[2-(2-methyl-5-nitro-1H-imidazol-1-Yl)­ethyl]­thio}­carbothioamide (**4a**)

To a round-bottom flask under an ice bath were added 0.300 g (1.07 mmol) of 1-(2-iodoethyl)-2-methyl-5-nitro-1H-imidazole (**2a**), 162 μL (2.13 mmol) of carbon disulfide, and, slowly, 301 μL (2.13 mmol) of *N*-ethyl-2-methyl-2-propanamine. The system was fitted with a calcium chloride drying tube and maintained under magnetic stirring in an ice bath for 30 min. The ice bath was then removed, and stirring was continued at room temperature for approximately 24 h.

Reaction progress was monitored by TLC (eluent: ethyl acetate/hexane 7:3; developer: iodine vapor). Upon completion, 25 mL of a 0.3 mol·L^–1^ hydrochloric acid solution were added, and the mixture was extracted with ethyl acetate (3 × 30 mL). The combined organic phases were washed with distilled water (20 mL), dried over sodium sulfate, filtered, and the solvent was removed under reduced pressure using a rotary evaporator.

The resulting solid was purified by flash column chromatography, using a hexane/ethyl acetate mixture as the mobile phase with the following gradient: 8:2, 7:3, and 6:4. Fractions containing the product were combined, yielding 0.126 g (36% yield) of **4a** as a yellowish solid. Mp: 94.5–95.0 °C. IR, ṽ/cm^–1^: (ATR) 3014, 2929, 1527, 1456, (1376, 1262, 1185.^1^ H NMR (400 MHz, acetone-d_6_), δ (ppm): 7.89 (1H, s, H-1), 4.67 (2H, t, *J*_5,6_ = 6.4 Hz, H-5), 4.07 (2H, q, *J*_12,13_ = 6.8 Hz, H-12), 3.74 (2H, t, *J*_6,5_ = 6.4 Hz, H-6), 2.51 (3H, s, H-4), 1.77 (9H, s, H-9, H-10, and H-11), 1.29 (3H, t, *J*_13,12_ = 6.8 Hz, H-13).^1,3^ C NMR (100 MHz, acetone-d_6_) and DEPT-135, δ (ppm): 195.00 (C, C-7), 152.19 (C, C-3), 133.57 (CH, C-1), 65.51 (C, C-8), 46.62 (CH_2_, C-5 or C-12), 45.40 (CH_2_, C-12 or C-5), 35.94 (CH_2_, C-6), 28.30 (3 × CH_3_, C-9, C-10, and C-11), 15.44 (CH_3_, C-13 or C-4), 14.62 (CH_3_, C-4 or C-13).

#### 2.4.2 Synthesis of 2-(2-Methyl-5-nitro-1H-imidazol-1-yl)­ethyl pyrrolidine-1-carboxylate (**7a**)

To a two-necked round-bottom flask, with one neck stoppered and under magnetic stirring, were added 0.595 g (1.94 mmol) of 3-methyl-1-(pyrrolidine-1-carbonyl)-1*H*-imidazol-3-ium iodide (**6a**), 0.332 g (1.94 mmol) of metronidazole, and 0.088 g (2.19 mmol) of sodium hydride (60% dispersion in mineral oil). After the addition of the reagents, the second neck of the flask was fitted with an anhydrous calcium chloride drying tube, and 15 mL of anhydrous tetrahydrofuran (THF) and 5 mL of anhydrous DMF were added via syringe and needle. The reaction mixture was maintained under magnetic stirring at room temperature for 24 h. Reaction progress was monitored by TLC (eluent: ethyl acetate/methanol 9:1; developer: iodine vapor). Upon completion, 20 mL of diethyl ether were added, and the mixture was extracted with distilled water (3 × 10 mL). The organic phase was dried over sodium sulfate, filtered, and the solvent was removed under reduced pressure using a rotary evaporator. The resulting oil was purified by flash column chromatography, using dichloromethane with increasing concentrations of methanol as the mobile phase. The following methanol percentages were employed: 1%, 2.5%, 5%, and 7.5%. The fractions containing the product were combined to afford 0.045 g of **7a** as a yellow oil (9% yield). IR (ATR), ν̅/cm^–1^: 2875, 1691, 1523, 1417, 1367, 1185. ^1^H NMR (400 MHz, CDCl_3_), δ/ppm: 7.96 (1H, s, H-1), 4.62 (2H, t, *J*_6,5_ = 5.2 Hz, 2 × H-6), 4.40 (2H, t, *J*_5,6_ = 5.2 Hz, 2 × H-5), 3.39–3.34 (4H, m, 2 × H-8, 2 × H-8′), 2.50 (3H, s, 3 × H-4), 1.89–1.80 (4H, m, 2 × H-9, 2 × H-9′).^1,3^ C NMR (100 MHz, CDCl_3_) and DEPT-135, δ/ppm: 153.91 (C, C-7), 150.79 (C, C-3), 138.52 (C, C-2), 133.03 (CH, C-1), 62.88 (CH_2_, C-6), 47.82 (2 × CH_2_, C-8, C-8′), 45.38 (CH_2_, C-5), 25.63 (2 × CH_2_, C-9, C-9′), and 14.12 (CH_3_, C-4).

### 2.5 *In Vitro* Assays

Following the virtual screening process, three compounds classified as active against *T. vaginalis*-resistant strains were selected for *in vitro* assays to evaluate their toxicity and activity against two strains. These experiments were conducted to enable a direct comparison between the outcomes predicted by the activity models and the results obtained from *in vitro* assays.

#### 2.5.1 Parasite and Cell Culture

Trophozoites CDC085 (ATCC 50143) and JT strains of *T. vaginalis* were cultured for 48 h at 37 °C (under anaerobic conditions) in Diamond’s trypticase yeast extract-maltose (TYM) medium supplemented with 10% (v/v) fetal-inactivated bovine serum,^45^ corresponding to the logarithmic growth phase. Parasites were grown to a density of 2 × 10^5^ cells mL^–1^. No antibiotics were used in culture.

#### 2.5.2 Anti-*Trichomonas vaginalis* assay and IC_50_ Determination

The three compounds were dissolved in dimethyl sulfoxide (DMSO, with the final concentration never exceeding 0.6% [v/v]). Anti-*T. vaginalis* activity was determined using 96-well microtiter plates. 9-fold serial dilution (maximum concentration of 100 μM) was incubated for 24 h at 37 °C in 5% CO_2_ atmosphere, containing 1 × 10^5^ trophozoites/mL. Parasites were counted using Trypan Blue 0.4% (1:1 [v/v]) in a hemocytometer to assess the trophozoites’ motility, morphology, and cell viability. IC_50_ values (the concentration of drug required for 50% inhibition) for the three compounds were determined using the GraphPad Prism v. 8.0.2 software package. Three controls were used: (i) positive control, metronidazole (200 μM for CDC085 and 4 μM for JT strains); (ii) negative control, no treatment; and (iii) vehicle control with DMSO 0.6%. All experiments were performed independently at least twice, in triplicate.

#### 2.5.3 Cytotoxicity against Human Cervical Adenocarcinoma Epithelial Cells

Human cervical adenocarcinoma epithelial cells (HeLa, CCL-2, BCRJ0100) were cultured in Dulbecco’s Modified Eagle Medium (DMEM) supplemented with 10% fetal-inactivated bovine serum. Cells were incubated at 37 °C in 5% CO_2_ atmosphere until a confluent cell monolayer was reached. HeLa cells were seeded in 96-well plates and treated with 400, 200, 100, 50, 25, and 12.5 μM of the compounds for 48 h at 37 °C in a 5% CO_2_ atmosphere. Cell viability was assessed by the 3-(4,5-dimethylthiazol-2-yl)-2,5-diphenyltetrazolium bromide (MTT) assay. The culture medium was removed, and the cells were treated with MTT dye at a concentration of 0.5 mg/mL and incubated for 4 h. The optical density of the solution was measured using a spectrophotometer at 570 nm. All experiments were performed independently in triplicate. Data were analyzed using the GraphPad Prism v. 8.0.2 software package to determine cytotoxic concentration 50% values (CC_50_). Negative (cells with no treatment) and vehicle (DMSO 2%) controls were used. The Selectivity Index (SI) was calculated according to the formula SI = CC_50_ Hela cells/IC_50_ *T. vaginalis*, considering values ≥10 as selective to the parasite.^46^

## 3 Results and Discussion

### 3.1 Generation of Machine Learning Models, Selection of the Most Predictive Ones, and Virtual Screening

Initially, ML-based QSAR models were generated for the prediction of *T. vaginalis* activities against susceptible and resistant strains, as well as host cytotoxicity against HeLa cells. These models are helpful for providing structural insights into molecular mechanisms related to those activities and could be applied to virtually screen novel and potential compounds for bioactivity in experimental profiling. The very first step of this work was obtaining datasets for modeling.

The datasets binarized for classification are unbalanced (susceptible and resistant datasets have ratios of 87/13 and 24/76 for active/inactive, respectively, and cytotoxicity has a 68/32 ratio for cytotoxic/noncytotoxic ratio, Table S1 and Figure S1). Therefore, we trained models using both unbalanced datasets and datasets balanced with the SMOTE oversampling strategy, as successfully reported in the literature.^34^

The first comparison of overall results suggests that SMOTE balancing strategies produced the most robust and predictive classification models for the three datasets (Figure 2). The analysis of average Matthews Correlation Coefficient (avgMCC, Figure 2A–C) values, as well as individual 5-fold cross-validation and external validation MCC values (cvMCC and extMCC, respectively, Figure 2D–F) evidence that models generated with unbalanced data have lower coefficients than models generated with balanced data. Therefore, all subsequent analyses were conducted only with data collected from models generated using SMOTE-balanced data.

**Figure 2:**
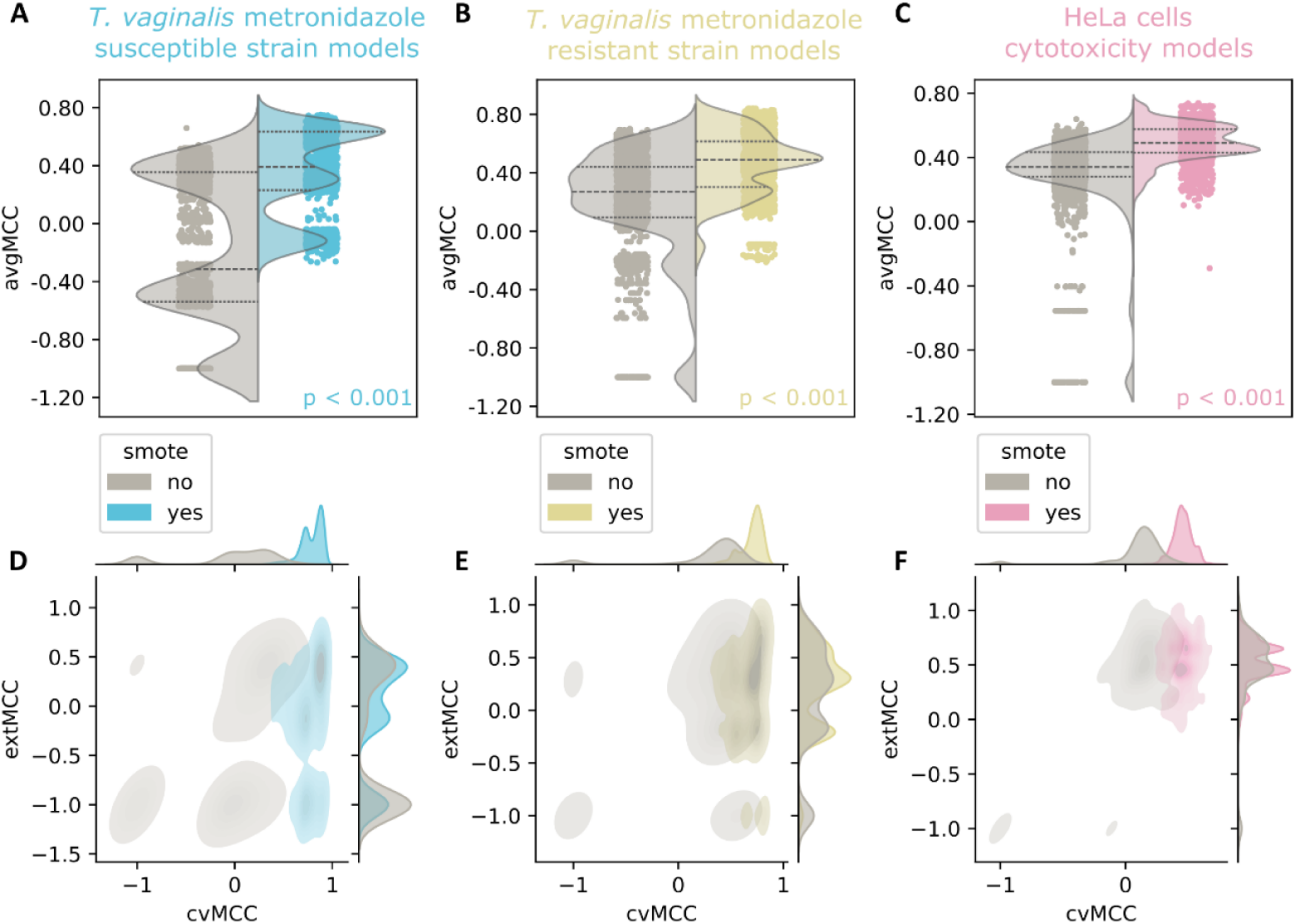
Violin plots for average MCC values for *T. vaginalis* metronidazole-susceptible strain models (A), *T. vaginalis* metronidazole-resistant strain models (B), and HeLa cells cytotoxicity models (C) compare models generated with a balanced dataset using SMOTE (colored data) and an unbalanced dataset (gray data). The distribution of MCC values was calculated using 5-fold cross-validation and external validation for all three models (D–F), also comparing the balanced dataset (colored data) and the unbalanced dataset (gray data).

Interestingly, the multilayer perceptron method produced the most robust and predictive models for susceptible and resistant strain datasets (Figure 3A,C), but the random forest method produced the best models for the cytotoxicity dataset (Figure 3E). However, despite the average values of avgMCC for all the *T. vaginalis* susceptible strain models generated with other methods (k-nearest neighbors, decision trees, and random forest) being higher than those with neural networks, MLP produced models with the highest value coefficients. The analysis of different fingerprints used as descriptors for training susceptible and resistant dataset models with multilayer perceptron (Figure 3B,D) and cytotoxicity dataset models with random forest (Figure 3F) suggested that AtomPair 2D, AtomPair 2D count, and Klekota-Roth fingerprints produced the best models, respectively. Therefore, the decision and selection of an optimal model for predicting classification for susceptible, resistant, and cytotoxicity datasets followed this combination of methods and fingerprints. This information is corroborated by the analysis of individual 5-fold cross-validation and external validation Matthews correlation coefficient values (Supporting Information, Figure S2).

**Figure 3:**
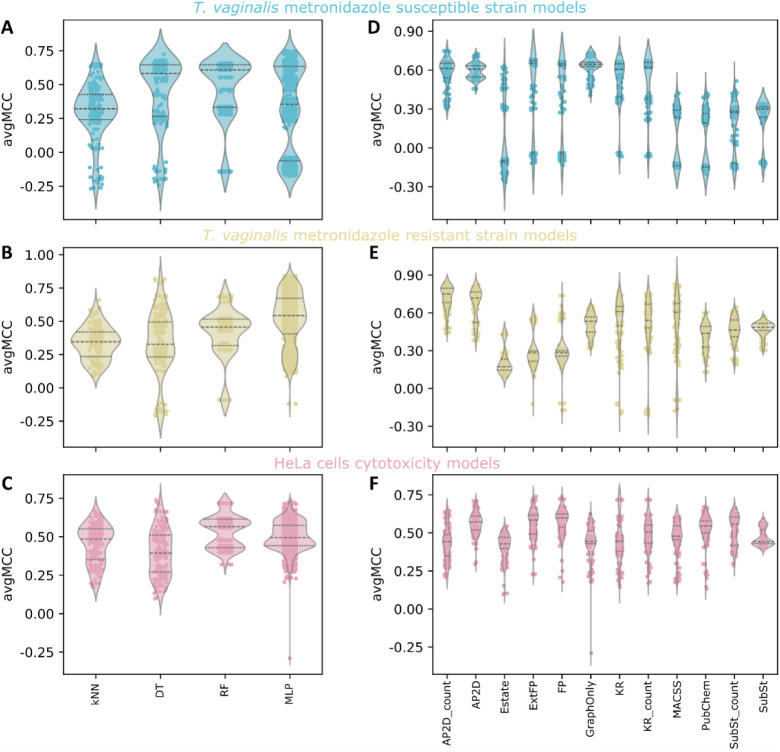
Violin plots for average MCC values for *T. vaginalis* metronidazole-susceptible strain models (A), *T. vaginalis* metronidazole-resistant strain models (B), and HeLa cells cytotoxicity models (C) comparing the machine learning methods used to generate them: kNN, DT, RF, and MLP. Similar violin plots for all three datasets comparing different fingerprint sets (AtomPair 2D count [AP2D_count], AtomPair 2D [AP2D], Estate, CDK Extended Fingerprint [ExtFP], CDK Fingerprint [FP], CDK graph-only fingerprint [GraphOnly], Klekota-Roth fingerprint [KR], Klekota-Roth count [KR_count], MACCS, PubChem, Substructure count [Subst_count], and Substructure fingerprint [Subst]) using MLP for both *T. vaginalis* datasets (D, E) and RF for cytotoxicity models (F).

As a last step of machine learning modeling, we selected an optimal model for each dataset. A Multilayer Perceptron model trained with AtomPair 2D fingerprint, 1 hidden layer, 5 neurons per layer, and 300 epochs generated the most predictive model for the susceptible dataset (Figure 4A). This model presented a 5-fold cross-validation Matthews Correlation Coefficient equal to 0.869 and an MCC calculated with the test set compounds equal to 0.583 (Figure 4B). Similarly, a Multilayer Perceptron model trained with AtomPair 2D count (nonbinary) fingerprint, 4 hidden layers, 20 neurons per layer, and 100 epochs generated the most predictive model for the resistant dataset (cvMCC and extMCC equal to 0.856 and 0.826, respectively, Figure 4C,D). Lastly, a Random Forest model trained with the Klekota-Roth fingerprint, maximum levels equal to 10, and 10,000 models generated the most predictive model for the cytotoxicity dataset (cvMCC and extMCC equal to 0.587 and 0.849, respectively, Figure 4E,F). Interestingly, in this case, Random Forest models did not vary in their performance with hyperparameter variation. Therefore, the simplest model was selected due to Occam’s razor rule. The obtainment of models with different methods, hyperparameters, and fingerprints is expected since there is no universal method reported to solve different tasks in drug design. Furthermore, different experimental models have their intrinsic relationship with chemical structures, which explains their own mechanisms heavily dependent on the dataset composition, size, and quality.^47^

**Figure 4:**
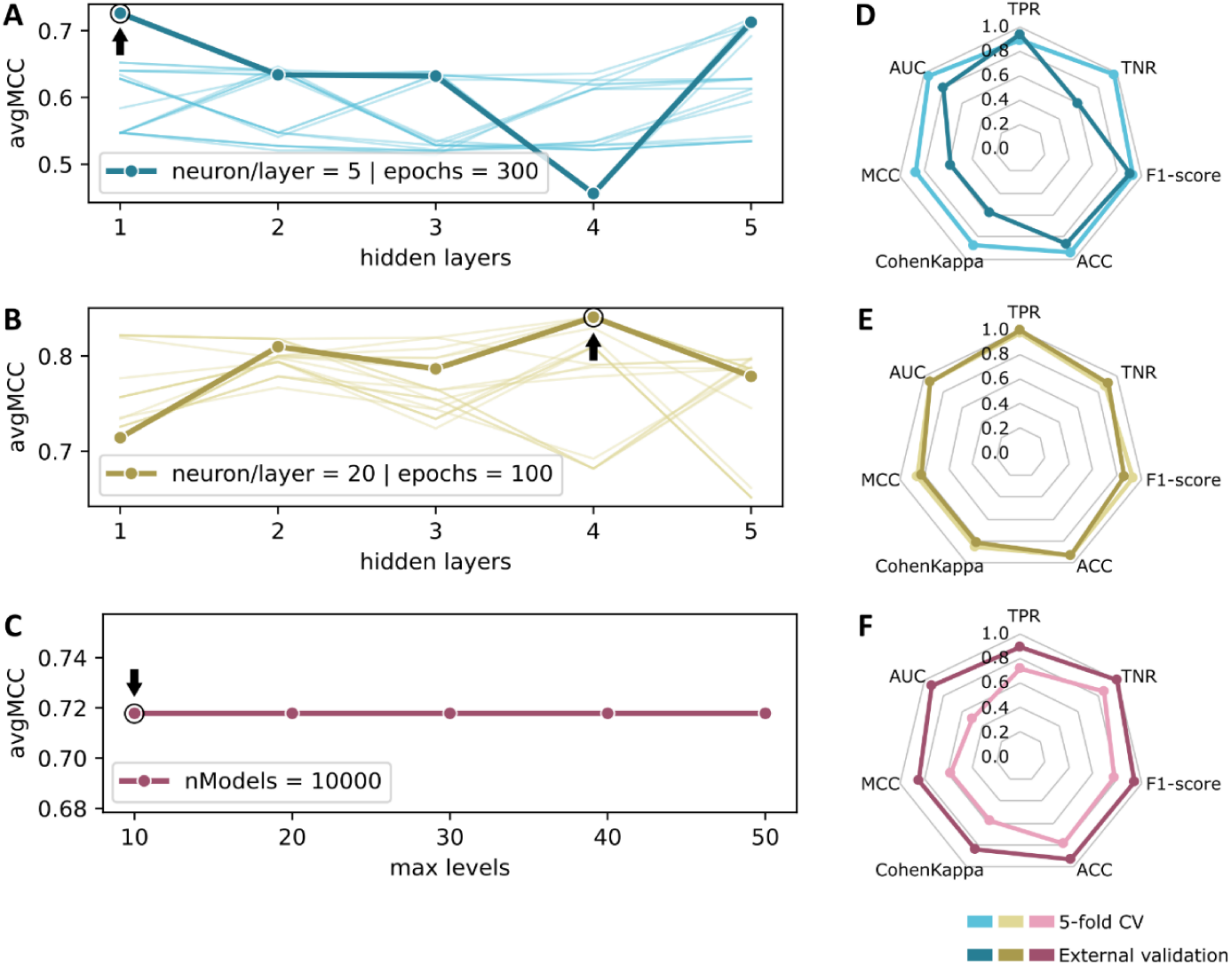
Line plot for the calculated average MCC values using models varying all hyperparameters for *T. vaginalis* metronidazole-susceptible strain models (A), *T. vaginalis* metronidazole-resistant strain models (B), and HeLa cells cytotoxicity models (C) comparing the machine learning methods used to generate them. Black arrows (A, B, and C) indicate the best set of hyperparameters used for the selection of the most predictive models. Radar plots for all metrics (true positive rate [TPR], true negative rate [TNR], F1-score, accuracy [ACC], Cohen Kappa, MCC, and area under the curve [AUC]) for all three selected models were generated using 5-fold cross-validation and external validation (D-F).

Although the models are reliable according to the literature standards for the application of QSAR models in drug design campaigns, we were aware that the datasets were unbalanced and the models could have some flaws. Specifically, the discrepancy between internal and external validation calculated metrics was observed for susceptible and cytotoxic models. Furthermore, the low amount of known data on the noncytotoxic compounds could bias the final model since this dataset is the smallest among the three studied endpoints. Before applying those three models in virtual screening, we interpreted them (Figure 5) according to the fifth principle of the Organization for Economic Co-operation and Development (OECD) guidelines for QSAR modeling.^48^

**Figure 5:**
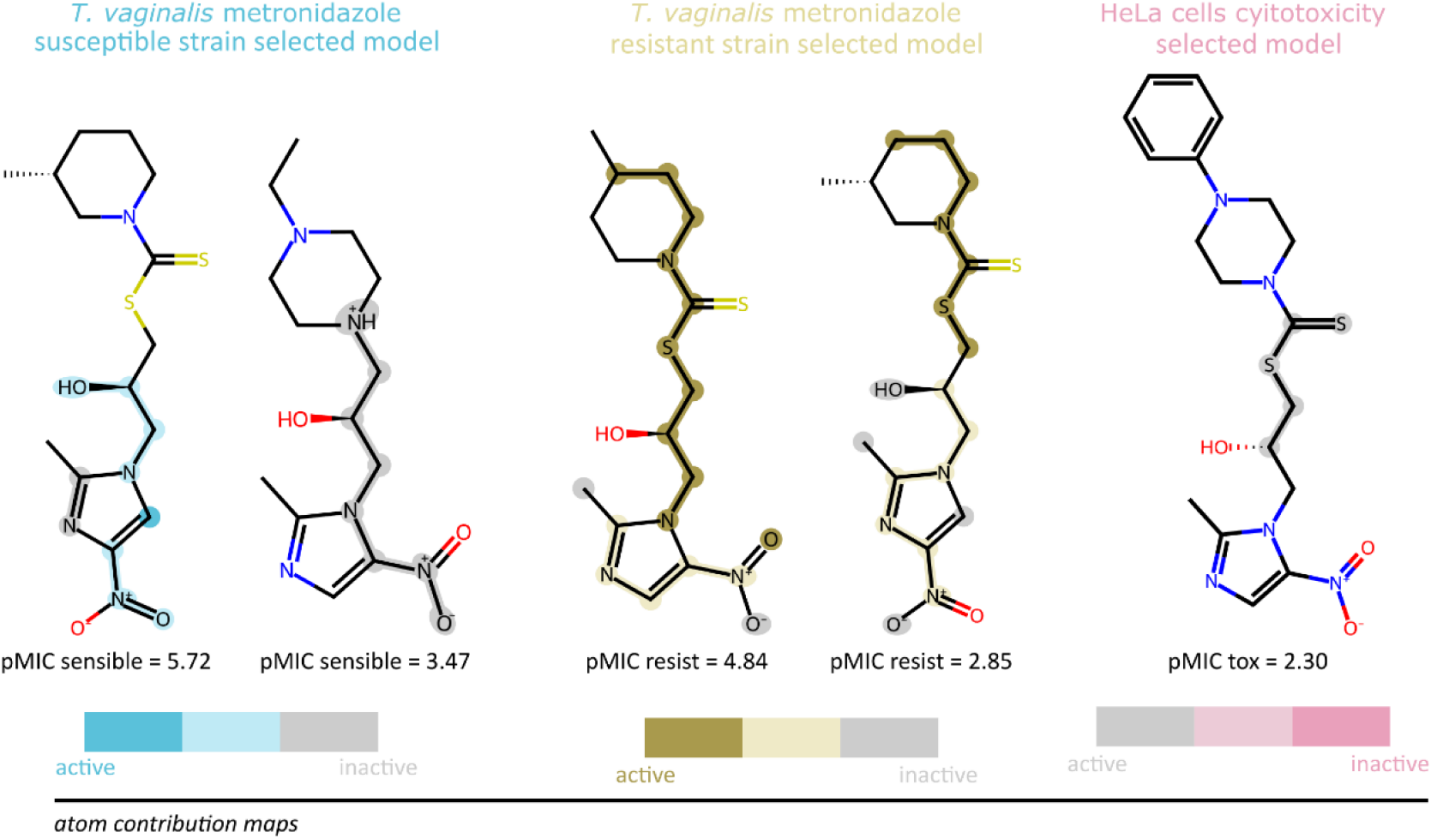
Atomic contribution maps for all the selected models. Colored atoms (dark teal, dark yellow, and dark pink) indicate the preferred groups to be kept due to their positive influence on anti-*T. vaginalis* and noncytotoxic activities. Middle colors (teal, yellow, and pink) indicate moderate positive influence on the activities. Gray atoms/groups negatively contribute to the three models.

Clearly, the presence of a nitro group at position 3 in the 5-methyl imidazole ring favors the activity for *T. vaginalis* susceptible strains, while the same group at position 2’ of the same ring favors the resistant strain. A hydroxyl group attached to the second carbon atom after the imidazole ring favors the susceptible strain activity, while the positively charged secondary amino group attached to an aliphatic chain disfavors the activity. Compounds against *T. vaginalis* metronidazole-resistant strains benefit from the dithiocarbamate group, while the hydroxyl group attached to the second carbon atom after the imidazole ring disfavors the activity. Lastly, the sulfur atom and the thiocarbonyl group of the dithiocarbamate moiety disfavor cytotoxicity, meaning they contribute to HeLa cell cytotoxicity.

After all models’ generation, comparison, validation, and selection of an optimum model for each dataset, followed by the models’ interpretation, we performed a virtual screening to select compounds for organic synthesis and experimental validation of this work. Two compounds (hits **3a** and **4a**, Table 1) were selected, prioritizing the nitro group at position 2 of the methyl-imidazole ring and the presence of a dithiocarbamate moiety, aiming for activity against *T. vaginalis* metronidazole-resistant strain. We also selected one compound with a carbamate group (hit **7a**, Table 1) for direct comparison against hit **3a**, as the only difference between the two molecules is the substitution of the two sulfur atoms with oxygen atoms in this functional group. All three hits were predicted to be cytotoxic, but we decided to proceed with the synthesis since the interpretation of classification models suggested that the groups responsible for cytotoxicity overlap (at least partially) with the groups responsible for anti-*T. vaginalis* activities. More importantly, there was a high bias favoring cytotoxic compounds in this specific dataset. Interestingly, the three hits (**3a, 4a,** and **7a**) were considered to fall within the applicability domain of all three selected models (Figure 6A–C). Prediction probabilities against both *T. vaginalis* strains were equal to 1 for the three hit compounds; however, these are classification predictions as active (IC_50_ lower than 50 μM). Lastly, calculations of HOMO and LUMO energies, as well as their differences, were performed as indicators of potential reduction susceptibility (as previously discussed in previous work^19^) and the hit compounds (**3a, 4a,** and **7a**) were comparable to control active compounds (Figure 6D).

**Table 1:**
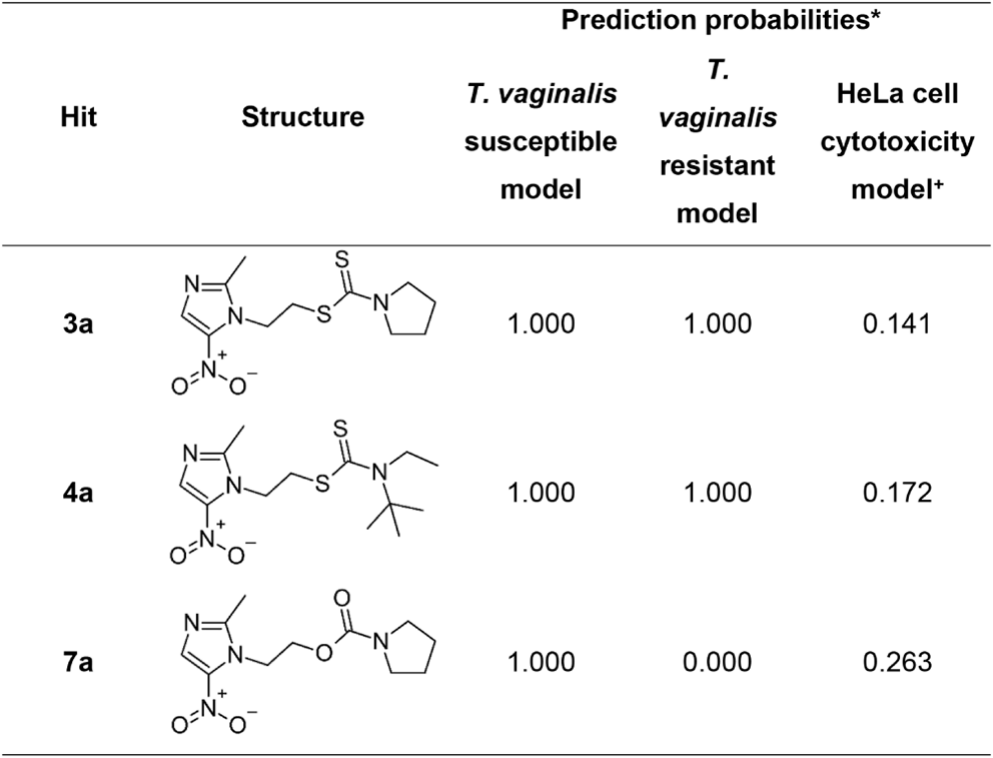
Molecular Structures of the Three Selected Hits as well as the Probability Values of Prediction for Each of the Three Selected Models

* Probabilities ranging from 0 to 1.

= Values equal to 1 mean the probability of being noncytotoxic.

**Figure 6:**
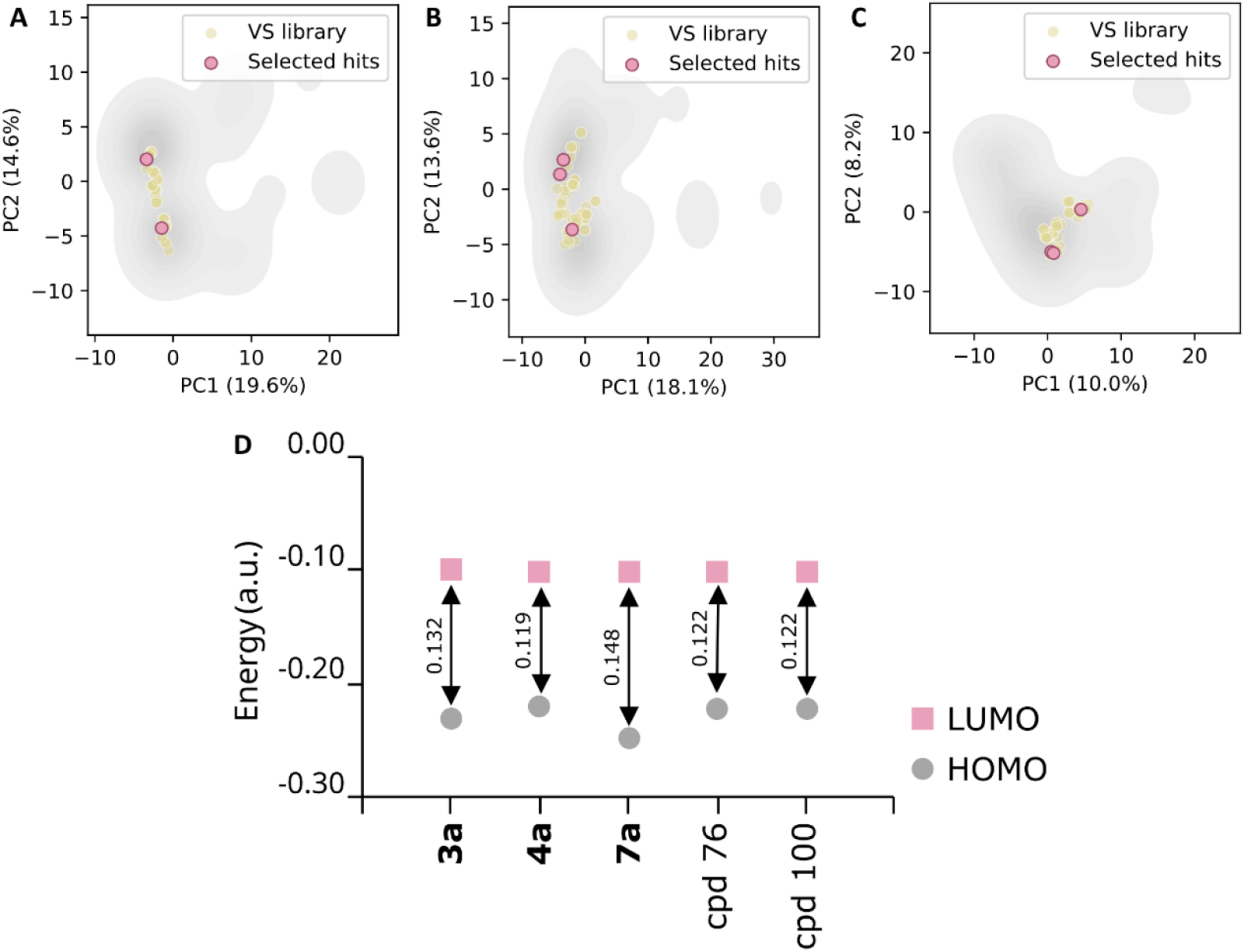
Principal component analysis (PCA) bidimensional projections created using the same fingerprint sets and training set compounds (gray density plot) for the three selected models (*T. vaginalis* metronidazole-susceptible strain [A], *T. vaginalis* metronidazole-resistant strain [B], and HeLa cell cytotoxicity model [C]). The projection of the VS library (57 compounds, yellow dots) and selected hits (pink dots). Plots A, B, and C were used to reduce the dimensionality of the chemical space used in the predictions, as well as to measure the applicability domain for virtual screening. Calculations of HOMO and LUMO energies for three selected hits (**3a, 4a,** and **7a**) and control compounds (cpd 76 and cpd 100, which were also used for comparison in Veríssimo’s work^19^) (D).

### 3.2 Synthesis of Selected Compounds

After the selection of compounds **3a**, **4a**, and **7a** by virtual screening, their synthesis enabled the acquisition of samples for experimental validation. Nitroimidazole derivatives were initially proposed based on theoretical studies, and their synthetic accessibility was assessed by evaluating the feasibility of their preparation under the available laboratory conditions and reagents. On the basis of this criterion, three derivatives identified as more accessible were selected for synthesis and obtained with minor adaptations to the reported procedures when necessary. The synthetic routes are illustrated in Scheme 1.

**Scheme 1:**
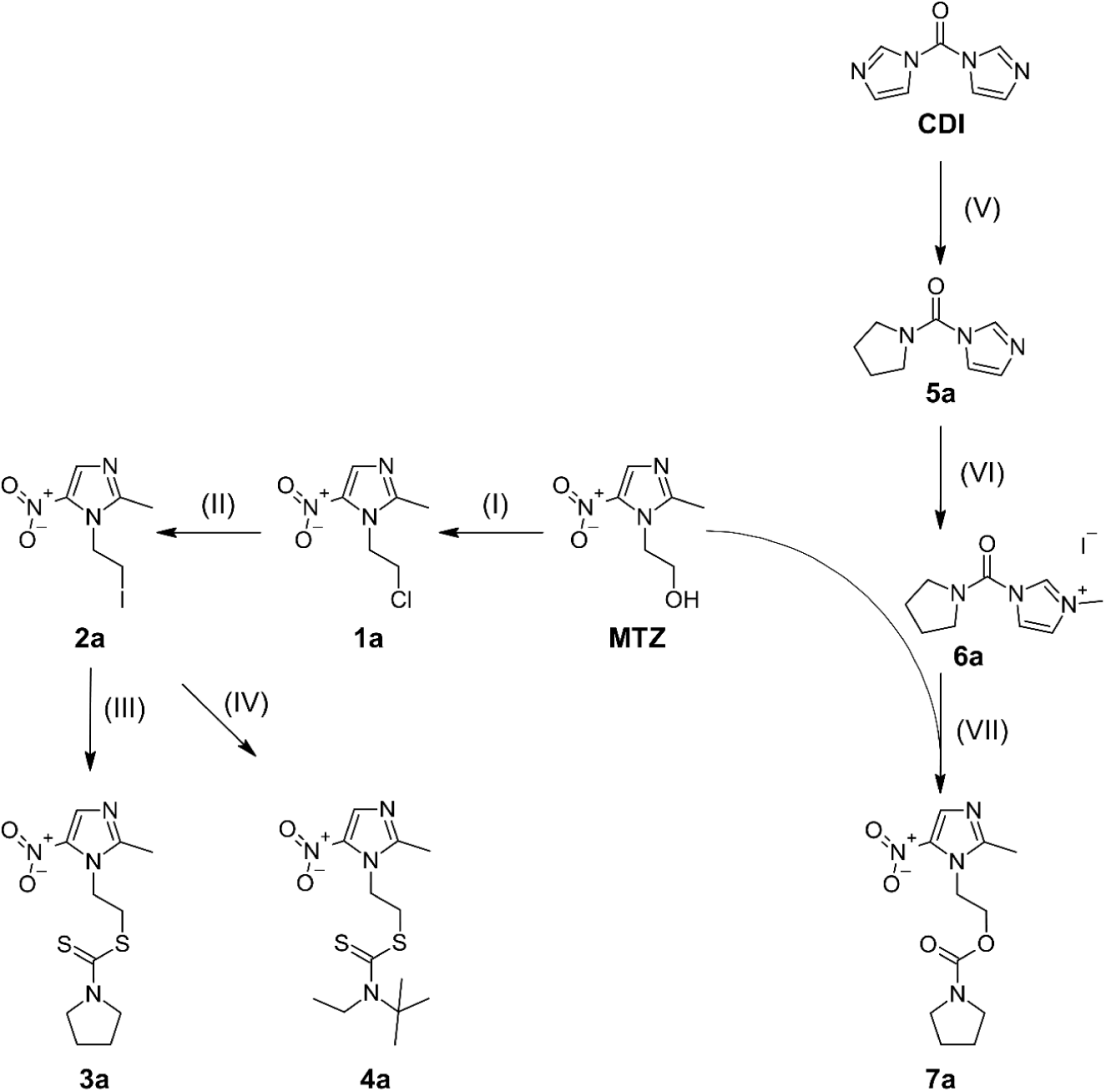
Reagents and Conditions^a^

a (I) Thionyl chloride, metronidazole, 70 °C, 3 h (94% yield); (II) **1a**, sodium iodide, *N*,*N*-dimethylformamide, 105 °C, 24 h (64% yield); (III) **2a**, carbon disulfide, pyrrolidine, room temperature, 24 h (36% yield); (IV) **2a**, carbon disulfide, *N*-ethyl-2-methyl-2-propanamine, room temperature, 24 h (36% yield); (V) 1,1′-carbonyldiimidazole (CDI), tetrahydrofuran, pyrrolidine, 60 °C, 16 h (70% yield); (VI) **5a**, acetonitrile, iodomethane, room temperature, 40 h (yield not determined; crude product used in the next step); (VII) **6a**, metronidazole (MTZ), sodium hydride, anhydrous tetrahydrofuran, anhydrous *N*,*N*-dimethylformamide, room temperature, 24 h (9% yield).

Dithiocarbamate derivatives **3a** and **4a** were obtained through a three-step procedure. Initially, MTZ was converted to the corresponding chlorinated intermediate **1a** by using thionyl chloride (SOCl_2_). Subsequently, nucleophilic substitution with excess sodium iodide afforded iodide intermediate **2a**. In the final step, this intermediate **2a** underwent nucleophilic substitution with carbon disulfide and secondary amines, yielding the desired dithiocarbamate derivatives **3a** and **4a**.

The carbamate derivative **7a** was also synthesized via a three-step procedure. First, 1,1’-carbonylimidazole (CDI) reacted with pyrrolidine via a nucleophilic acyl substitution (addition–elimination) to form the carbamoylimidazole intermediate **5a**, which was then methylated with iodomethane, affording 3-methyl-1-(pyrrolidine-1-carbonyl)-1H-imidazol-3-ium iodide **6a**. In the final step, MTZ was deprotonated by sodium hydride to generate the corresponding alkoxide, which subsequently attacked the carbonyl group of **6a**, resulting in the formation of carbamate **7a** via an addition–elimination mechanism and the release of the imidazole-derived leaving group.

### 3.3 *In Vitro* Assessment of Selected Compounds

Finally, after the synthesis and characterization of the hit compounds, the computational studies could be properly validated. Then, samples of compounds **3a**, **4a**, and **7a** were sent for experimental assays to confirm their anti-*T. vaginalis* and cytotoxic activities. The compounds’ effects on *T. vaginalis* trophozoites can be seen in Table 2 and Figure 7. The compounds have been shown to be highly promising against the JT *T. vaginalis* strain in *in vitro* tests. The toxic effect on the parasite was evaluated using the IC_50_, which showed values below 2 μM for all three compounds. Regarding the 3CDC085 strain, the **3a** compound proved to be the most promising, with excellent inhibitory activity (IC_50_ = 9.33 ± 0.8 μM). Compounds **4a** and **7a** demonstrated a moderate effect (IC_50_ = 48.92 ± 3.4 μM and 34.44 ± 2.4 μM, respectively). All three tested compounds showed IC_50_ values within the range of predictions to classify them as active compounds according to the machine learning models, thereby confirming the selection of hits by virtual screening. As expected, the metronidazole positive control showed inhibitory effects. Recent studies have shown that compounds with an IC_50_ of less than 10 μM possess excellent anti-*T. vaginalis* activity and are highly promising for further preclinical *in vitro* and *in vivo* testing.^49–51^

**Table 2:** Anti-*Trichomonas vaginalis* Activity and HeLa Cytotoxicity of Three Compounds **3a, 4a,** and **7a** ^a^ ^b^

	**Anti-** *Trichomonas vaginalis* **activity** **IC**_**50**_ **± SD (μM)**			**Selectivity index**	
Compound	JT strain	CDC085 strain	HeLa cytotoxicity CC50 ± SD (μM)	JT strain	CDC085 strain
**3a**	0.72 ± 0.1	9.33 ± 0.8	338.4 ± 25.2	470	36.3
**4a**	0.17 ± 0.0	48.92 ± 3.4	166.5 ± 11.0	976.5	3.4
**7a**	1.52 ± 0.1	34.44 ± 2.4	379.1 ± 25.7	249.3	14.8
**MTZ**	2.07 ± 3.6	26.25 ± 2.4	-		-

a SD: standard deviation.

b MTZ: metronidazole.

**Figure 7:**
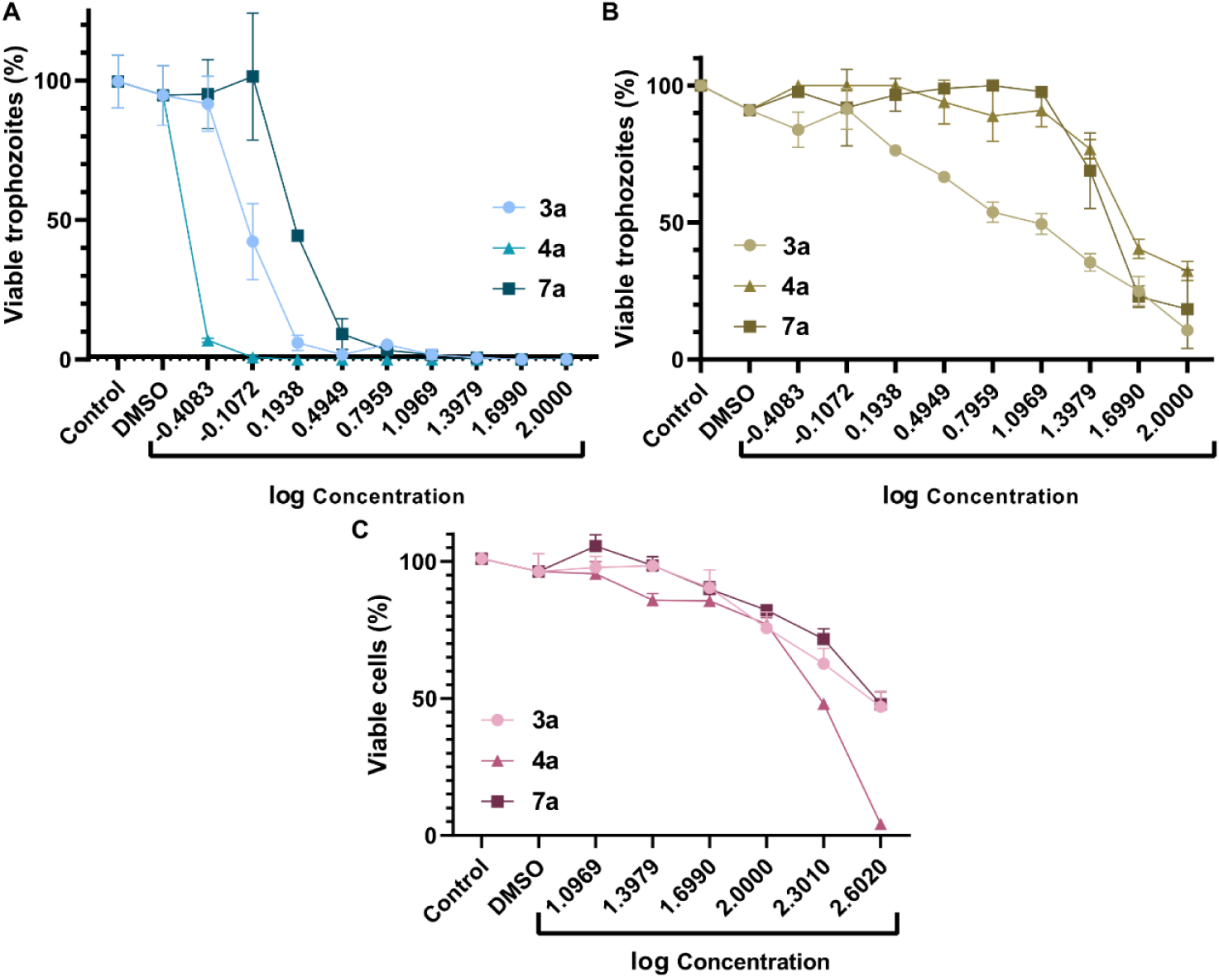
% Viability of JT (A) and CDC085 (B) *Trichomonas vaginalis* trophozoites and HeLa cells (C) after 48 h of exposure to compounds **3a**, **4a**, and **7a** at concentrations ranging from 0.396 to 100 μM (A and B) and 12.5 to 400 μM (C).

Although the search for novel drug candidates usually focuses on resistant microbial strains, finding alternatives for treating infections caused by metronidazole-susceptible *T. vaginalis* strains is also a valid objective. The marked heterogeneity of *T. vaginalis* strains^52,53^ and the genetic adaptability of metronidazole-resistant strains^54^—which influence the high virulence and pathogenicity of metronidazole-sensitive strains—allow us to explore different scenarios. Furthermore, considering that most infections are caused by susceptible strains, the development of therapeutic alternatives to nitroimidazoles is justified. In addition, it is known that prolonged use of metronidazole can cause adverse effects, such as central nervous system toxicity.^55,56^ Therapeutic alternatives based on different pharmacological classes could replace the use of this drug in routine clinical practice.

All compounds showed low *in vitro* toxicity against HeLa cells. In particular, the compounds **3a** and **7a** showed very low toxicity, with CC_50_ values greater than 300 μM (Table 1). When the selectivity index for the protozoan was evaluated, all compounds were highly selective for the JT *T. vaginalis* strain (SI > 249.3), and the **3a** and **7a** compounds were selective against the CDC085 *T. vaginalis* strain (SI = 36.3 and 14.8, respectively). For the compound **4a**, despite presenting a CC_50_ of 166.5 μM, it was not selective for the CDC085 *T. vaginalis* strain (SI = 3.4).

## 4 Conclusions

The combination of ML algorithms and different fingerprint sets allowed the proper classification of known nitroimidazole derivatives according to their anti-*T. vaginalis* activities against metronidazole-susceptible and -resistant strains, as well as their cytotoxicity against HeLa cells. Initially, due to the unbalanced nature of the three data sets used, the comparison of modeling with and without SMOTE (the oversampling technique used) allowed us to conclude that training ML models with balanced datasets generated more predictive and robust models. In this work, the MLP and RF methods produced the most predictive and selected models for the next steps, with external MCC values ranging from 0.583 to 0.849. The models were also interpreted using the variable exchange method in combination with statistical comparison of the features in active and inactive subsets. This interpretation suggested that the 5-methyl nitroimidazole ring is essential to the anti-*T. vaginalis* activity, while the presence of dithiocarbamate favors activity against resistant strains and HeLa cell toxicity. The models were used to virtually screen a designed library of nitroimidazole derivatives, and the three identified hits (**3a**, **4a**, and **7a**) were synthesized and evaluated against *T. vaginalis* and HeLa cells. All compounds presented activity against JT and 3CDC085 *T. vaginalis* strains, with IC_50_ values ranging from 0.17 to 48.92 μM, and no cytotoxicity for HeLa cells was observed at the tested concentrations, showing them to be promising candidates for preclinical evaluation.

## Supplementary Material





## Data Availability

We used OMEGA 2.5.1.4 and QUACPAC 1.6.3.1 for ligand preparation, distributed under an academic license. MASSA Algorithm v.0.9.6 and PaDEL (v1.0) are freely available. Jaguar (version 4, implemented in the Maestro 2024.3) is available from Schrödinger, LLC, New York, NY, 2024, under a commercial license. Third-party software employed in the manuscript includes GraphPad PRISM version 8.2 (https://www.graphpad.com/ and Schrödinger Suite 2024.3 (https://www.schrodinger.com) which are distributed under their respective licenses. The authors used ChatGPT (GTP-5.3 model) for language revision and grammar correction; the authors assume full responsibility for the present manuscript.

## Notes

(ZIP)
Table S1 contains the percentage of compounds in each class for all three datasets modeled in this work. Figure S1 also displays the percentage of compounds in each class for all three data sets modeled (*T. vaginalis* metronidazole-susceptible in blue shades, *T. vaginalis* metronidazole-resistant in yellow shades, and HeLa cytotoxicity in pink shades) in this work. Figure S2 shows 5-fold cross-validation and external validation MCC values for all models generated with different methods (left panel) and different fingerprints (right panel). Figures S3 to S7 correspond to ^1^H and ^13^C Nuclear Magnetic Resonance (NMR) spectra. An SDF molecular file with the dataset compounds, including classes (1 for actives, 0 for inactives, ND for no data) for *T. vaginalis* metronidazole-susceptible (susTv) and resistant (resTv) strains and HeLa cells (HeLa), as well as labels for training and test subsets (PDF)
