# Supplementary material for: Machine Learning Classification Models for the Design of Novel Nitroimidazole Derivatives with Anti-Trichomonas vaginalis Activity
Source: ACS Omega. 2026 Jul 23;11(30):45515–27. doi: 10.1021/acsomega.6c05649 (PMC13449211; doi:10.1021/acsomega.6c05649)

SUPPORTING INFORMATION

Machine learning classification models for the design of novel activities of nitroimidazoles derivatives with anti-Trichomonas vaginalis activities

Gabriel Corrêa Veríssimo[1,2], Anand Miranda Antoniassi[1], Laura Gonçalves Rezende[3,4], Victor Rocha Lamego[3,4], Wanderleya Toledo dos Santos[3,4], Ana Cláudia Chagas de Paula[3,4], Lauren Hubert Jaeger[3,4], Thales Kronenberger[5,6,7], Renata Barbosa Oliveira[1,*], Vinícius Gonçalves Maltarollo[1,*]

[1] Departamento de Produtos Farmacêuticos, Faculdade de Farmácia, Universidade Federal de Minas Gerais, Av. Antônio Carlos, 6627. Pampulha, Belo Horizonte, MG, 31270-901, Brazil.

[2] Postgraduate Programme in Bioinformatics, Federal University of Minas Gerais (UFMG), Belo Horizonte, Brazil.

[3] Programa de Pós-graduação em Ciências Farmacêuticas, Faculdade de Farmácia, Universidade Federal de Juiz de Fora, Rua José Lourenço Kelmer, s/n, campus universitário, São Pedro, Juiz de Fora - MG, 36036-900, Brazil.

[4] Laboratório de Células-Tronco e Parasitologia, Faculdade de Farmácia, Universidade Federal de Juiz de Fora, Rua José Lourenço Kelmer, s/n, campus universitário, São Pedro, Juiz de Fora - MG, 36036-900.

[5] Institute of Medical Microbiology and Hygiene, Interfaculty Institute of Microbiology and Infection Medicine, Eberhard Karls Universitat Tübingen.

[6] German Center for Infection Research (DZIF), partner-site Tübingen. Elfriede-Aulhorn-Str. 6, 72076 Tübingen, Germany.

[7] School of Pharmacy, Faculty of Health Sciences, University of Eastern Finland, Kuopio 70211, Finland.

*Corresponding authors: renatabo.ufmg@gmail.com; viniciusmaltarollo@gmail.com

Table S1. Percentual of compounds in each class for all the three datasets modeled in this work.

***T. vaginalis* metronidazole susceptible strain dataset**			
	**complete set**	**training set**	**test set**
**active**	86.67%	89.29%	76.19%
**inactive**	13.33%	10.71%	23.81%

***T. vaginalis* metronidazole resistant strain dataset**			
	**complete set**	**training set**	**test set**
**active**	23.68%	24.19%	21.43%
**inactive**	76.32%	75.81%	78.57%

**HeLa cells cytotoxicity dataset**			
	**complete set**	**training set**	**test set**
**cytotoxic**	67.95%	67.19%	71.43%
**non-cytotoxic**	32.05%	32.81%	28.57%

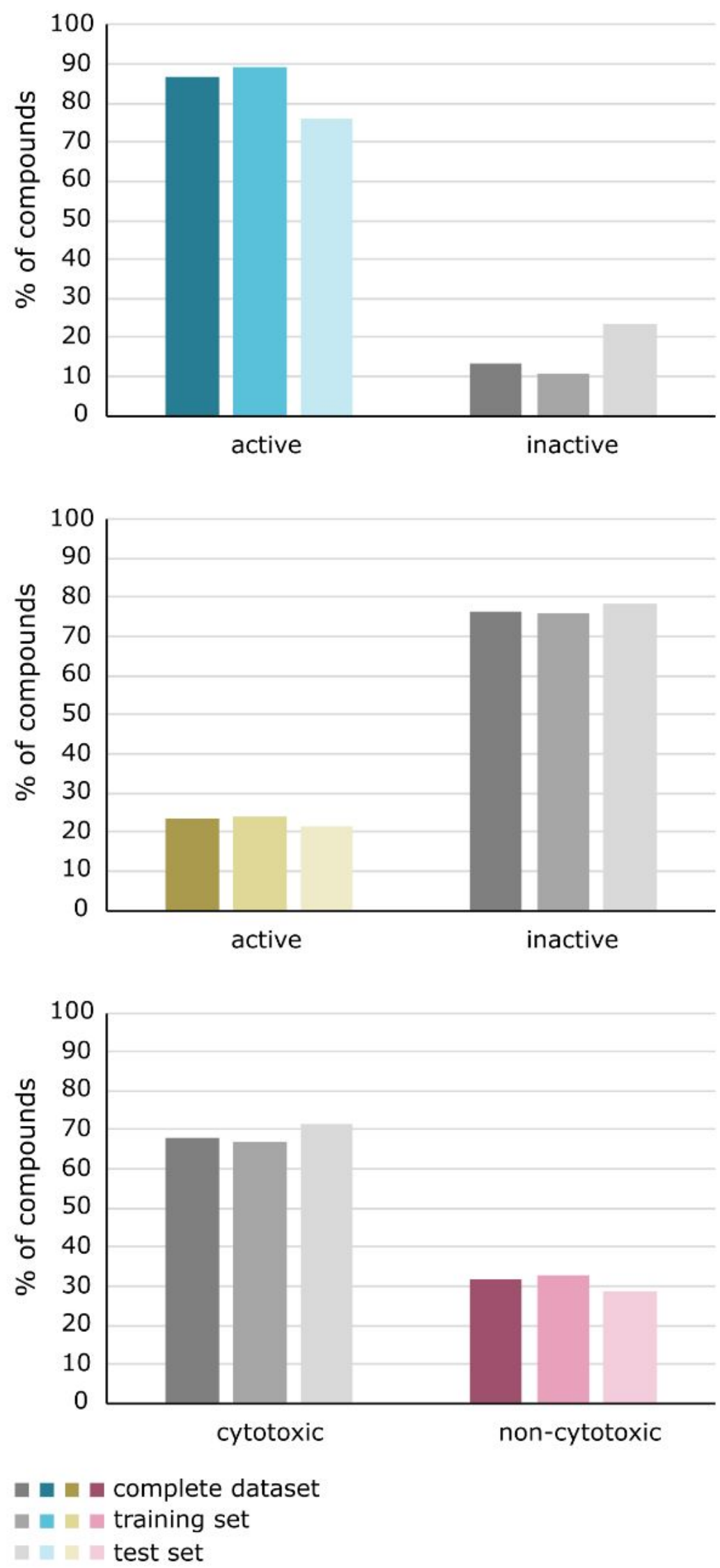

Figure S1. Percentual of compounds in each class for all the three datasets modeled (*T. vaginalis* metronidazole susceptible in blue shades, *T. vaginalis* metronidazole resistant in yellow shades, and HeLa cytotoxicity in pink shades) in this work.

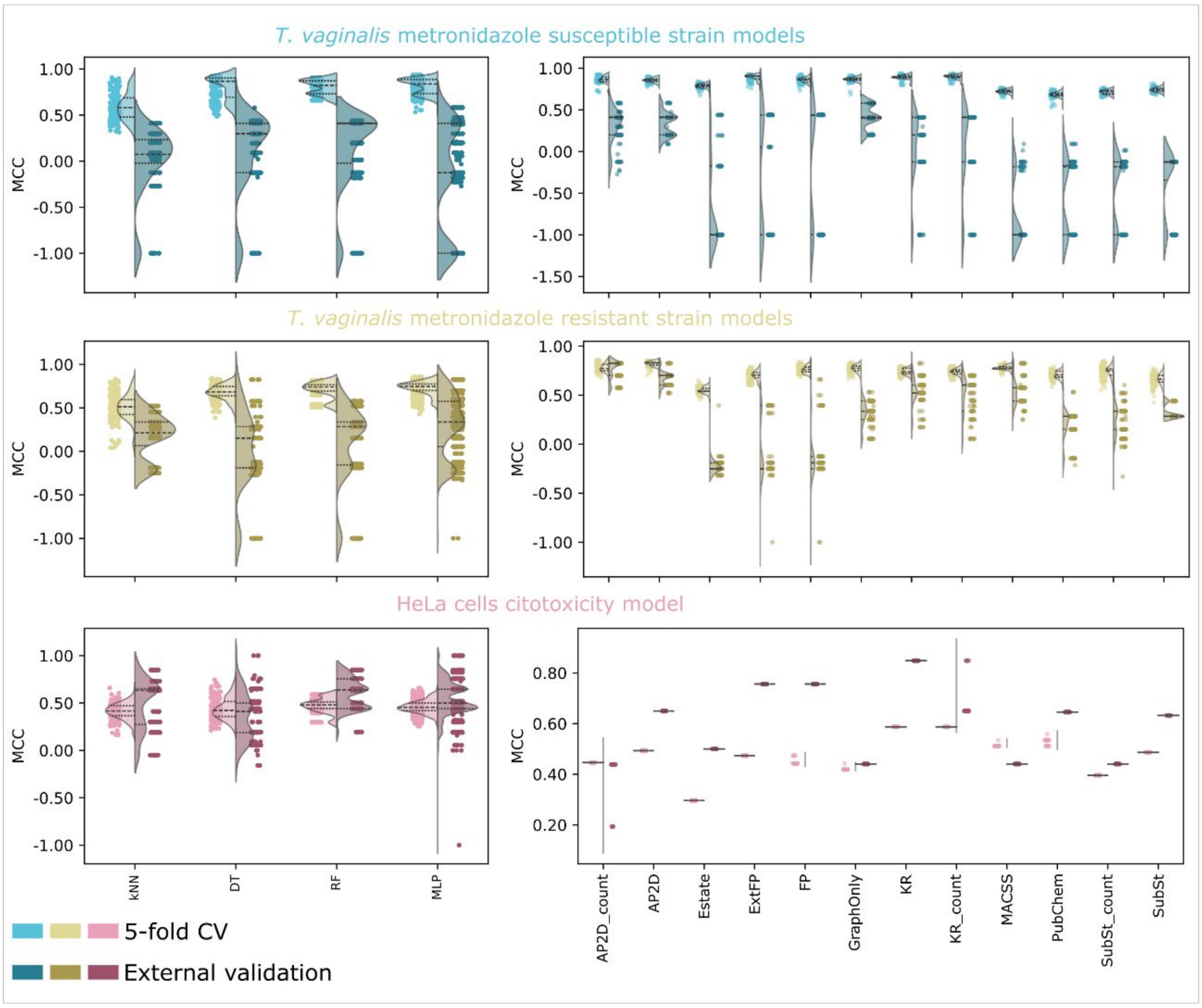

Figure S2. 5-fold cross-validation and external validation MCC values for all models generated with different methods (left panel) and different fingerprints (right panel).

^{1}H and [13]C Nuclear Magnetic Resonance (NMR) spectra

Figure S3. Infrared spectrum of compound 1a

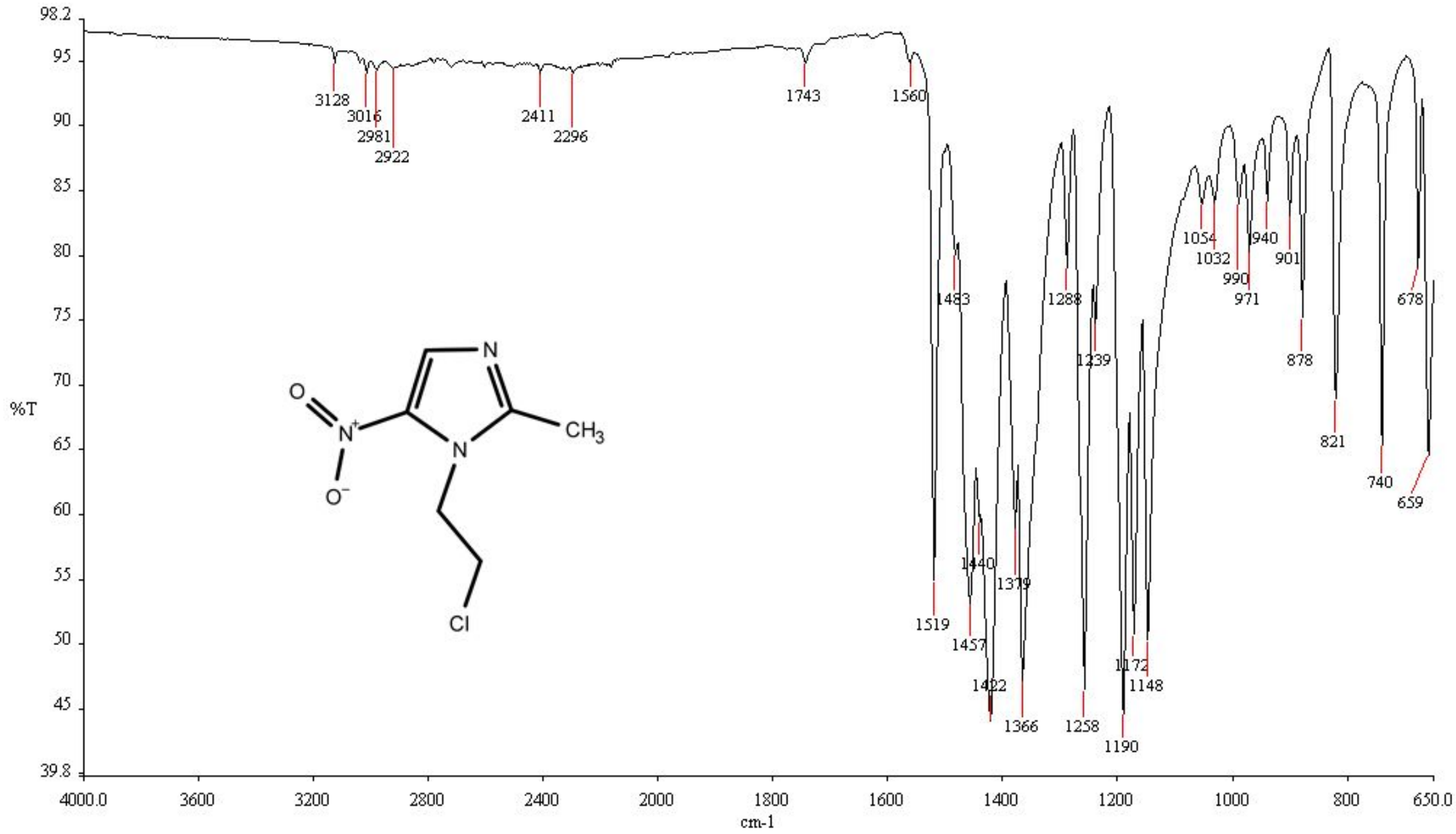

Figure S4. ^{1}H NMR (400 MHz, $CDCl_3$) spectrum of compound 1a

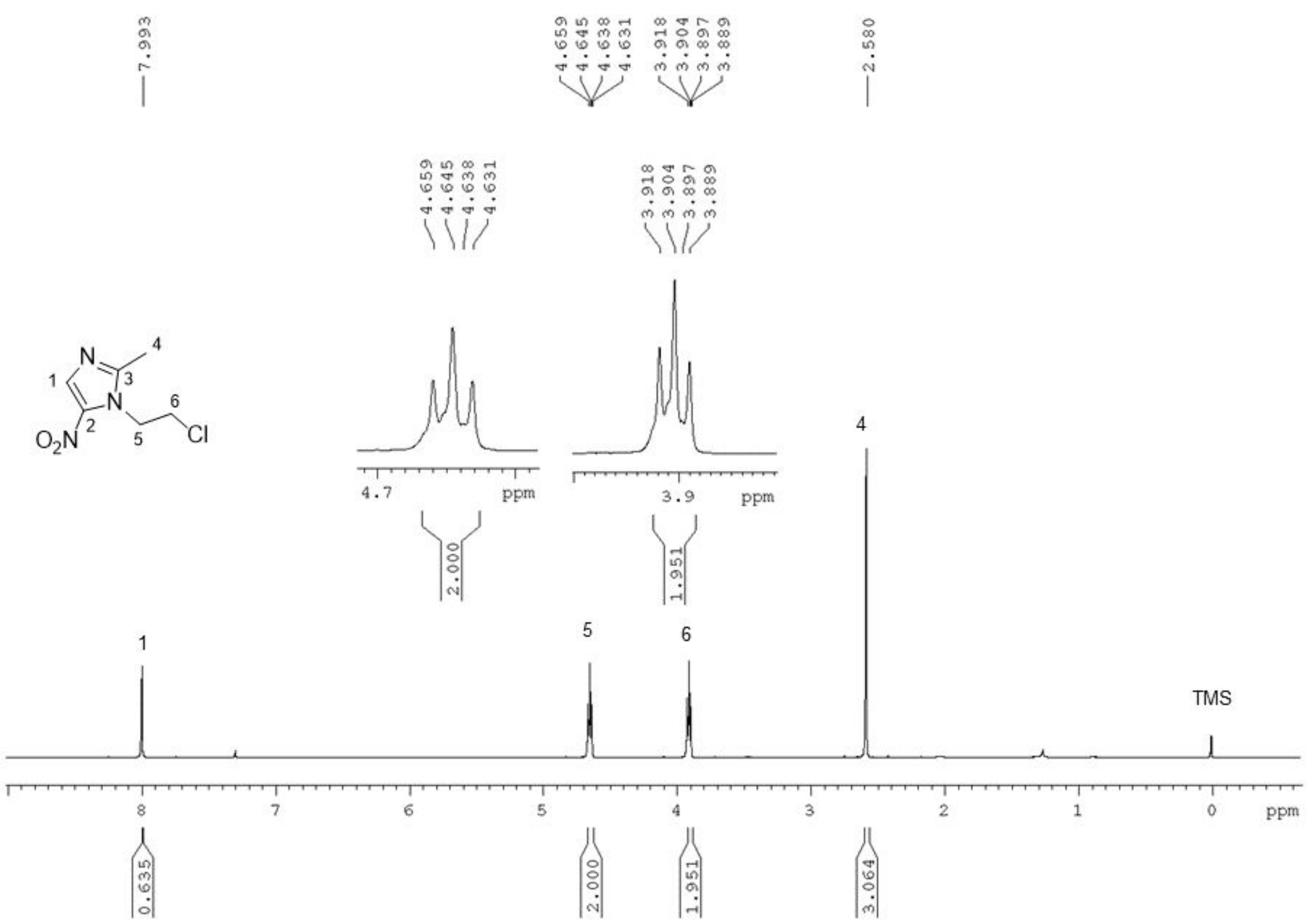

Figure S5.^{13}C and DEPT-135 NMR (100 MHz, $CDCl_3$) spectra of compound 1a

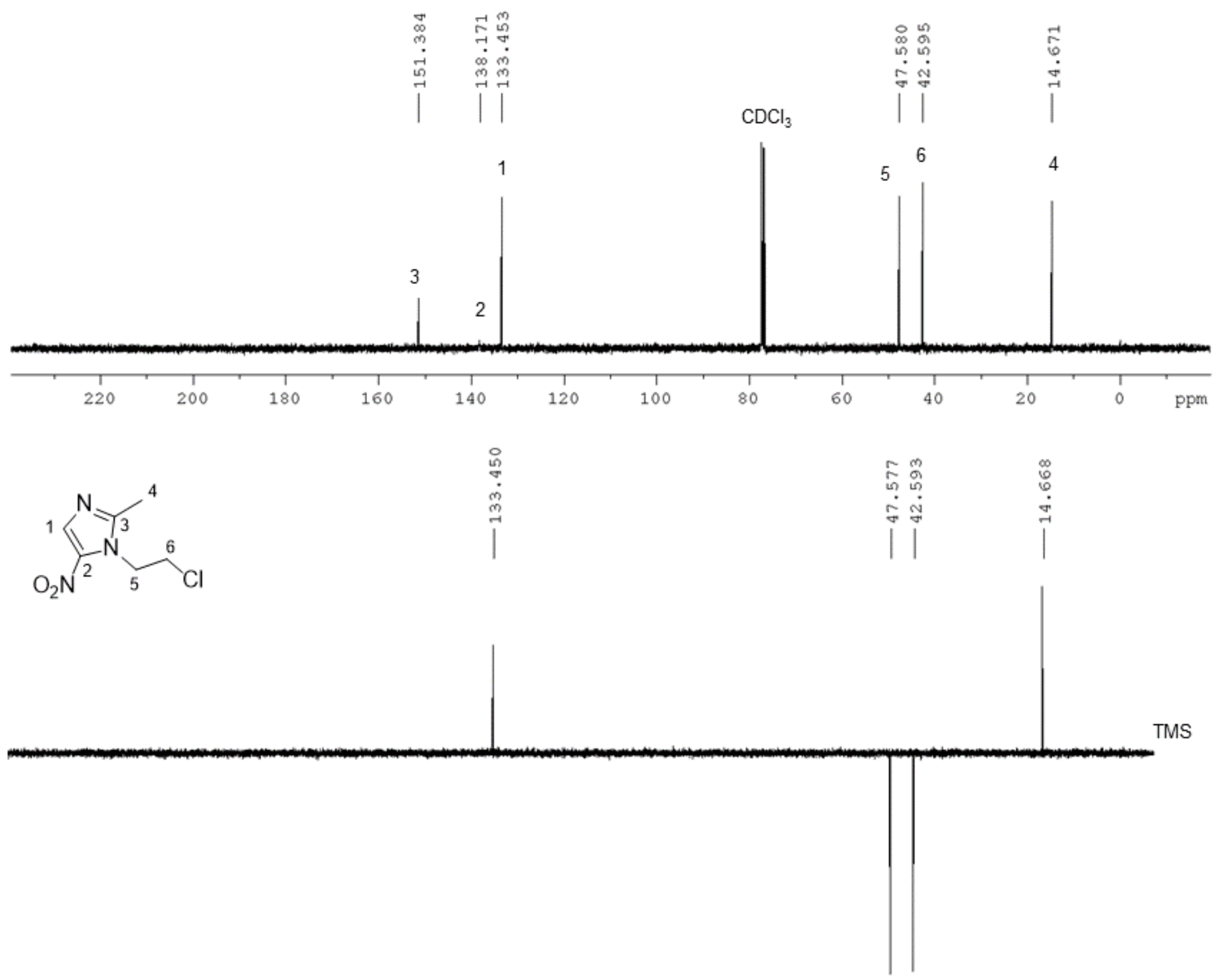

Figure S6. Infrared spectrum of compound 2a

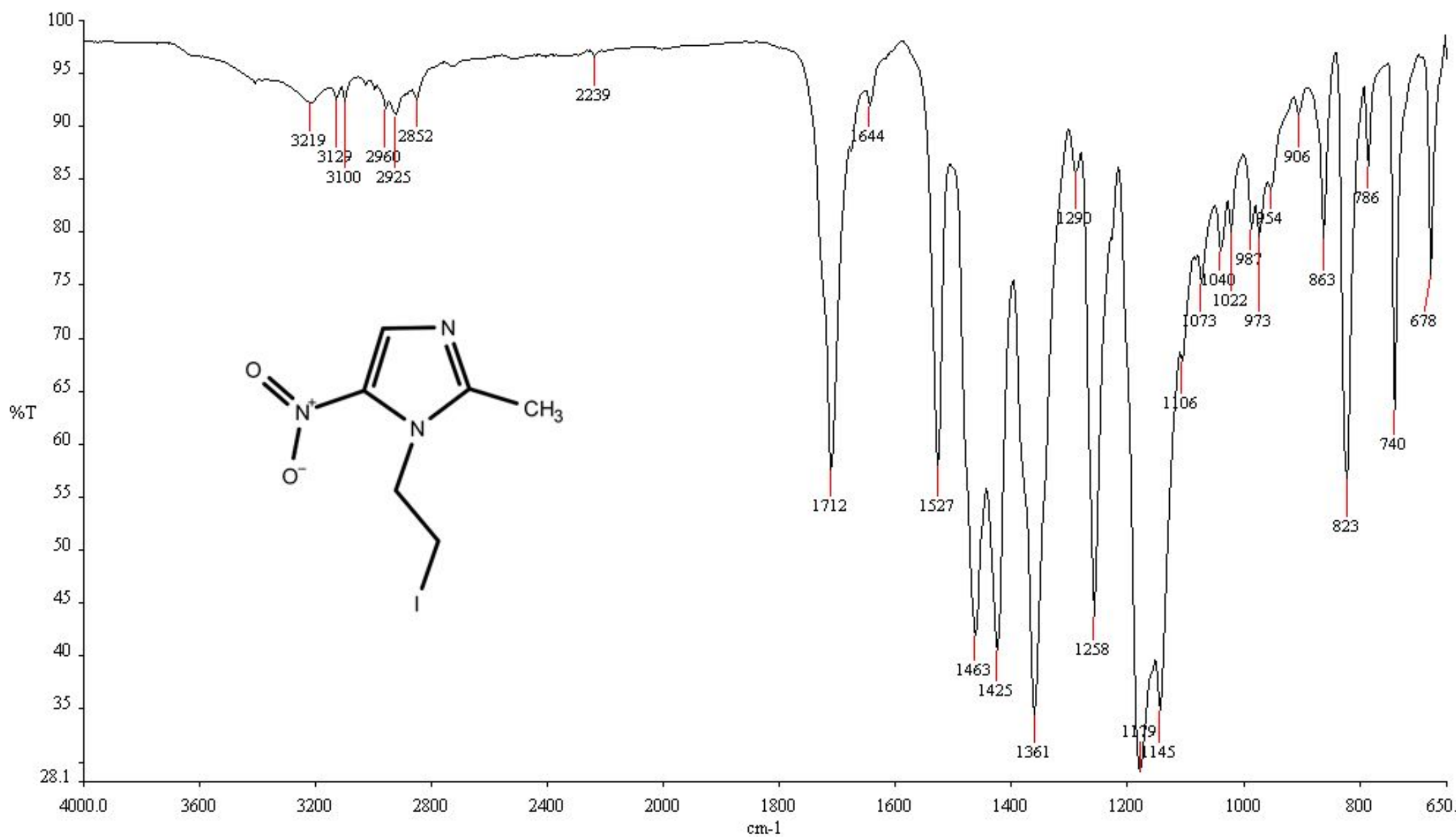

Figure S7.^{1}H NMR (400 MHz, acetone-d_6) spectrum of compound 2a

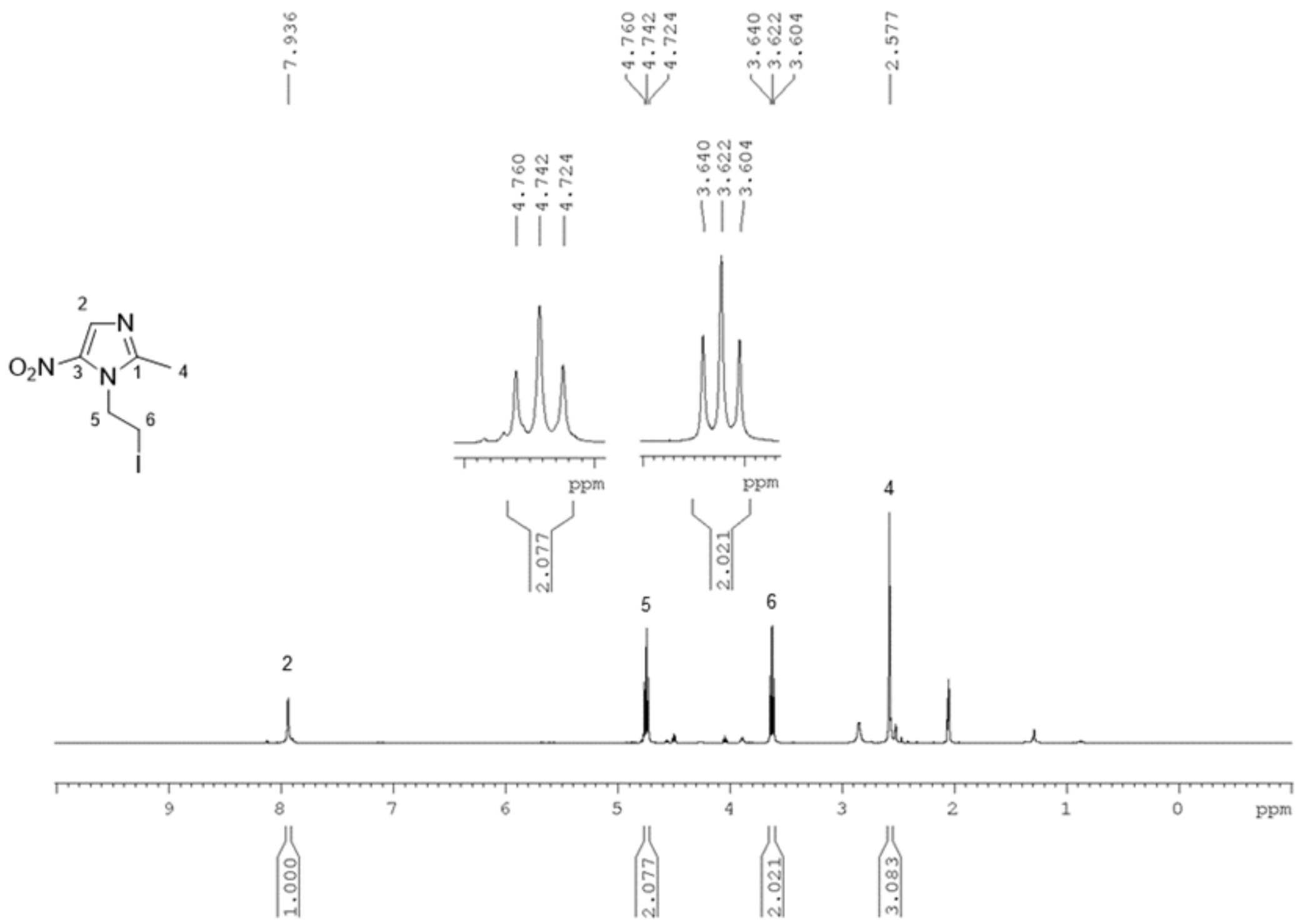

Figure S8. ^{13}C NMR (100 MHz, acetone-d_6) spectra of compound 2a

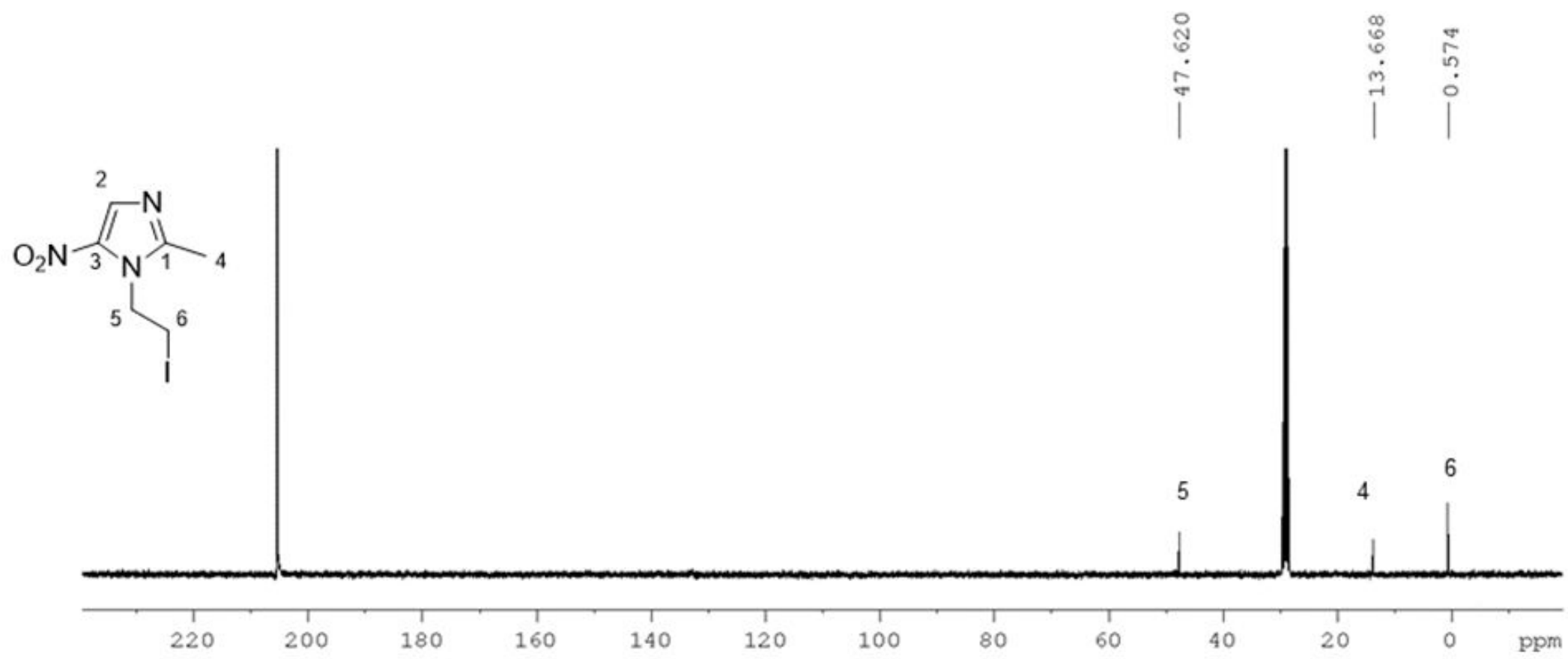

Figure S9. Infrared spectrum of compound 3a

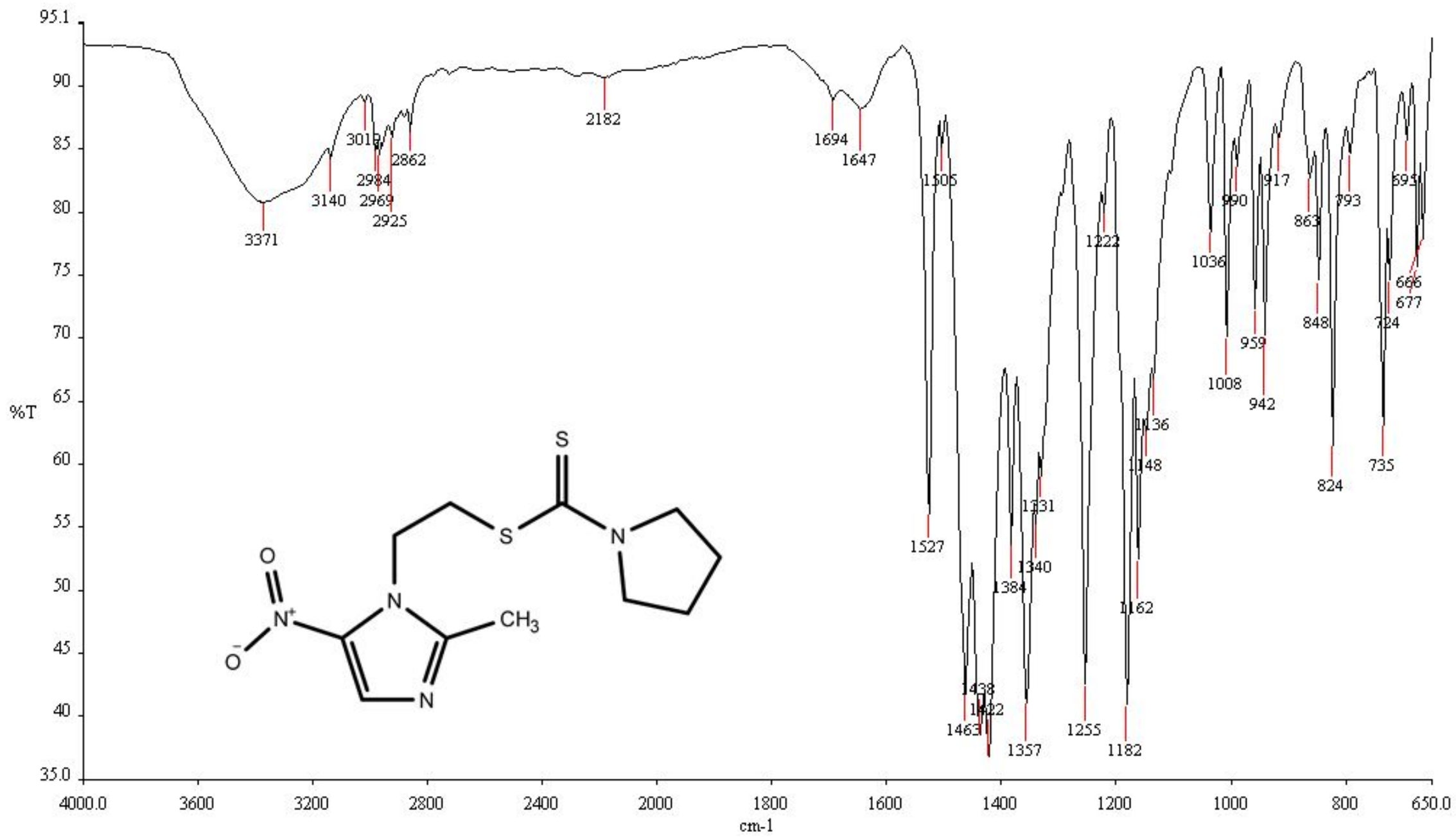

Figure S10.^{1}H NMR (400 MHz, $CDCl_3$) spectrum of compound 3a

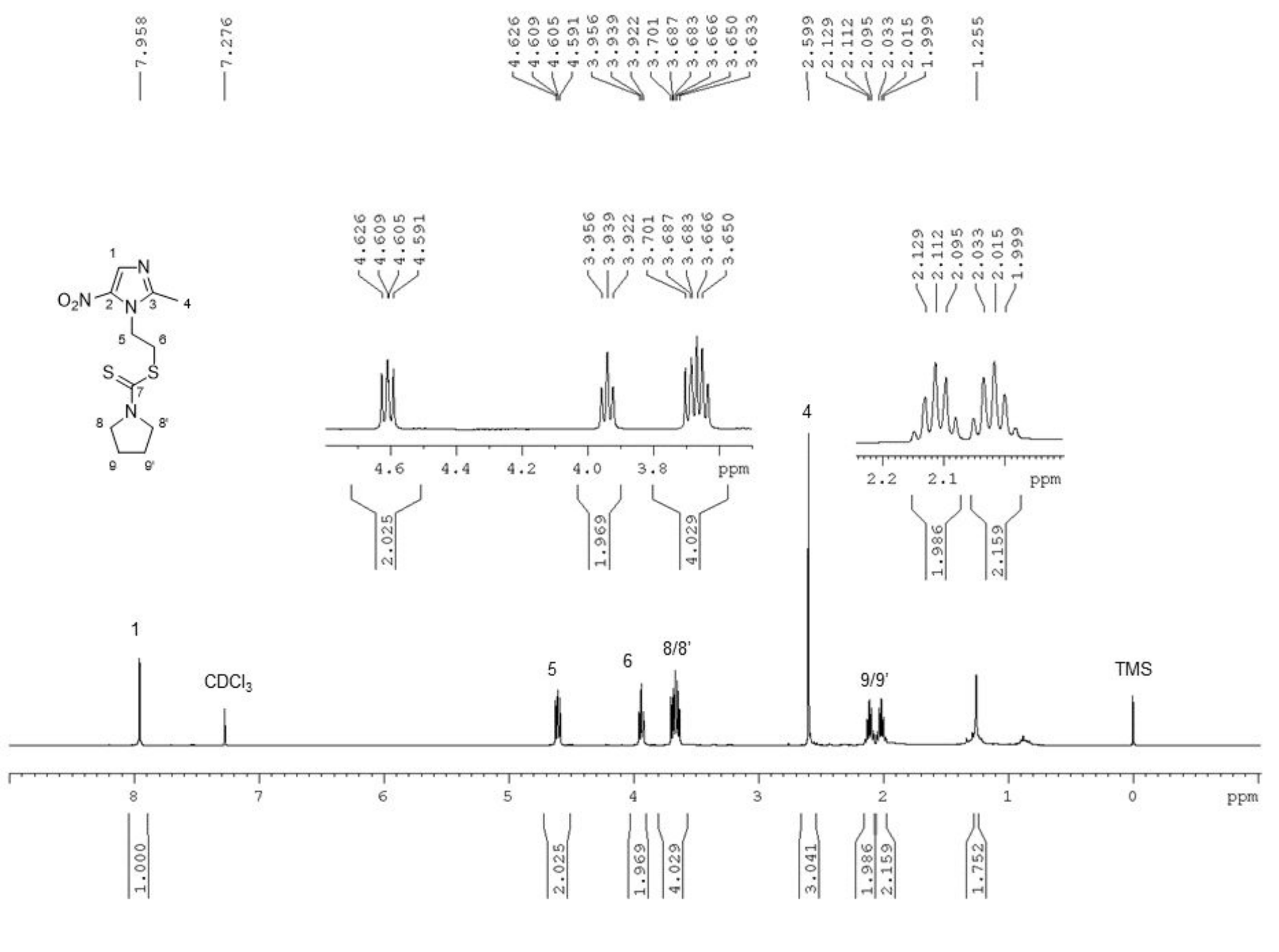

Figure S11. ^{13}C and DEPT-135 NMR (100 MHz, $CDCl_3$) spectra of compound 3a

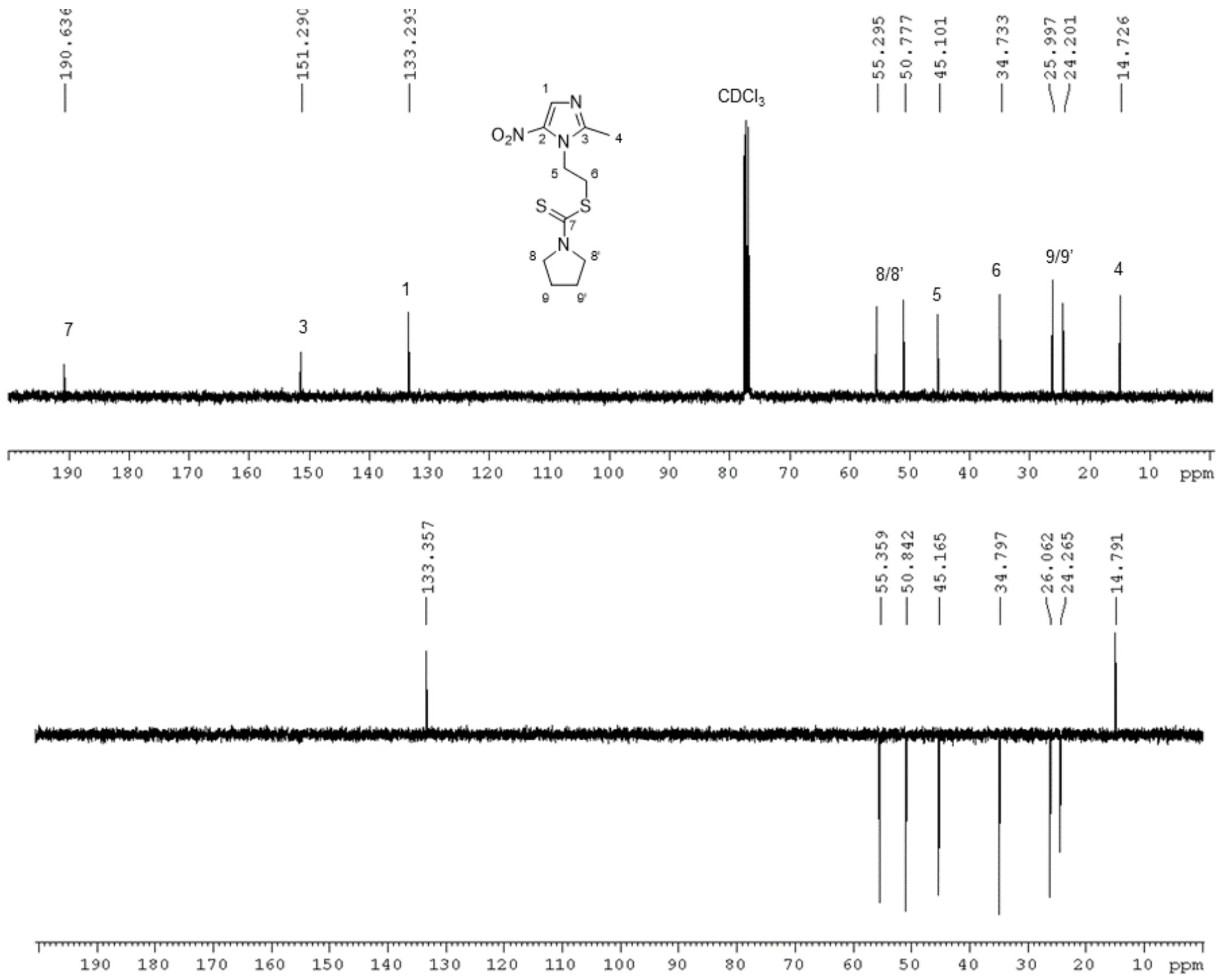

Figure S12. Infrared spectrum of compound 4a

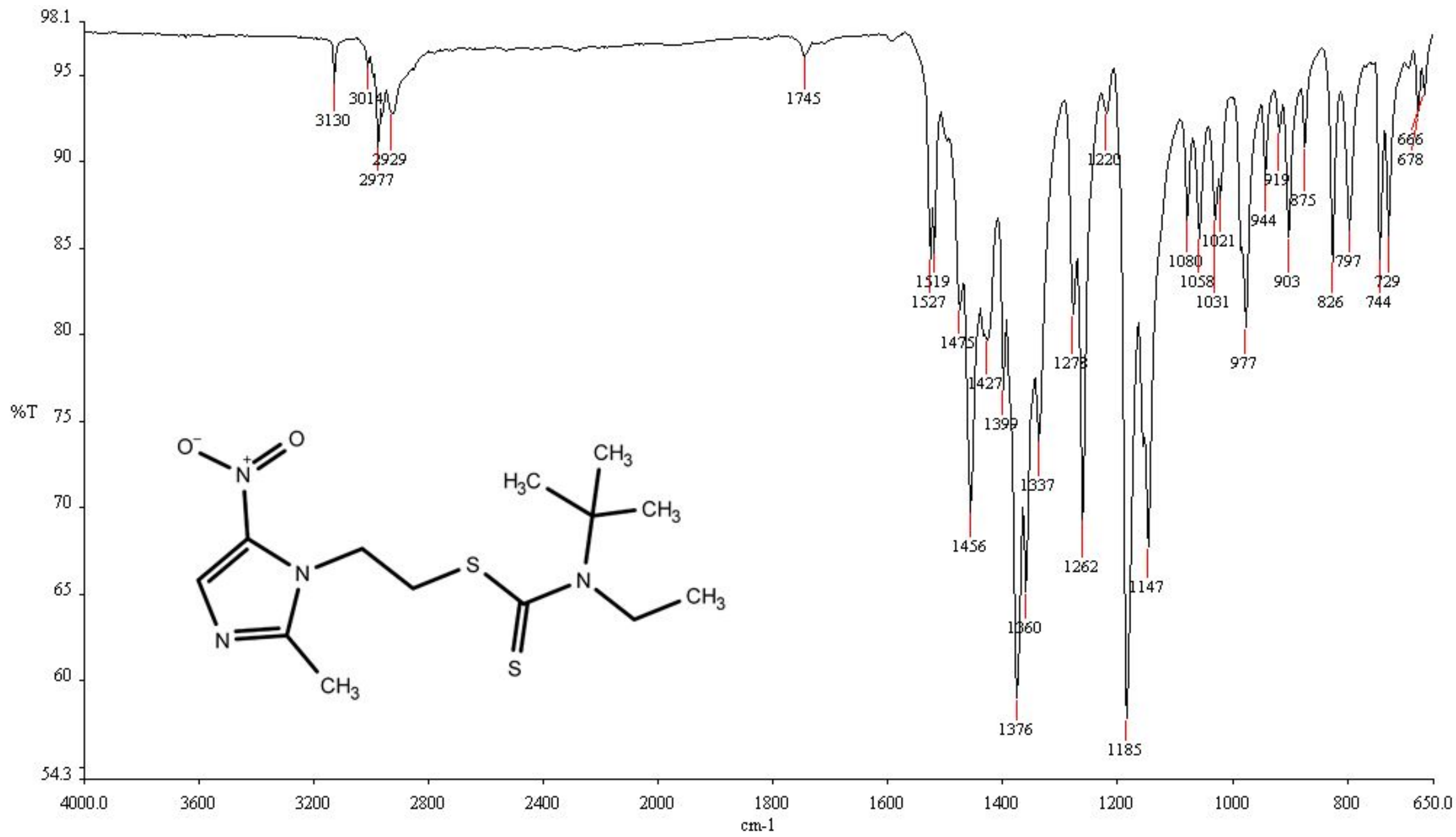

Figure S13.^{1}H NMR (400 MHz, acetone-*d*$_6$) spectrum of compound 4a

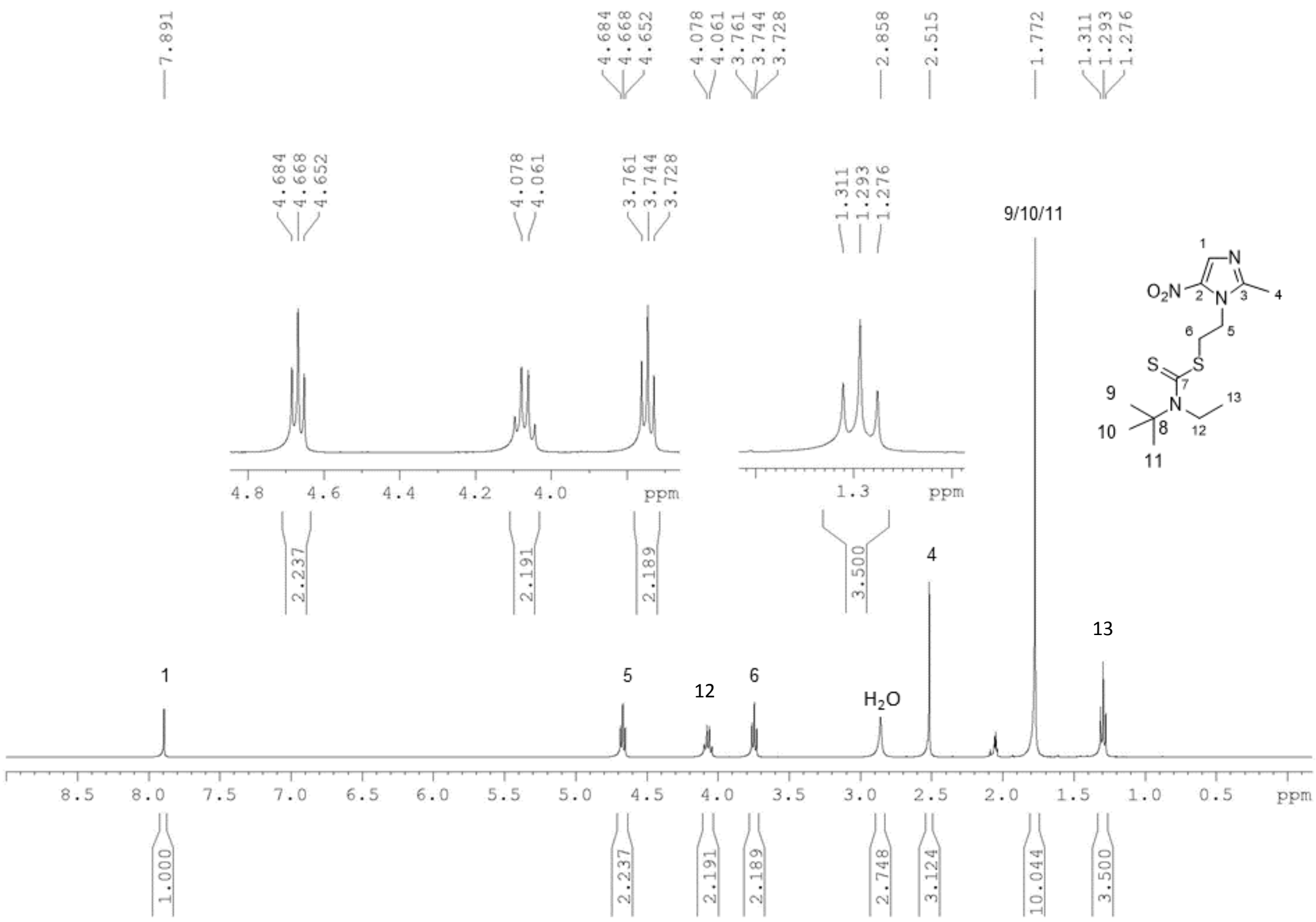

Figure S14.^{13}C and DEPT-135 NMR (100 MHz, acetone-*d*$_6$) spectra of compound 4a

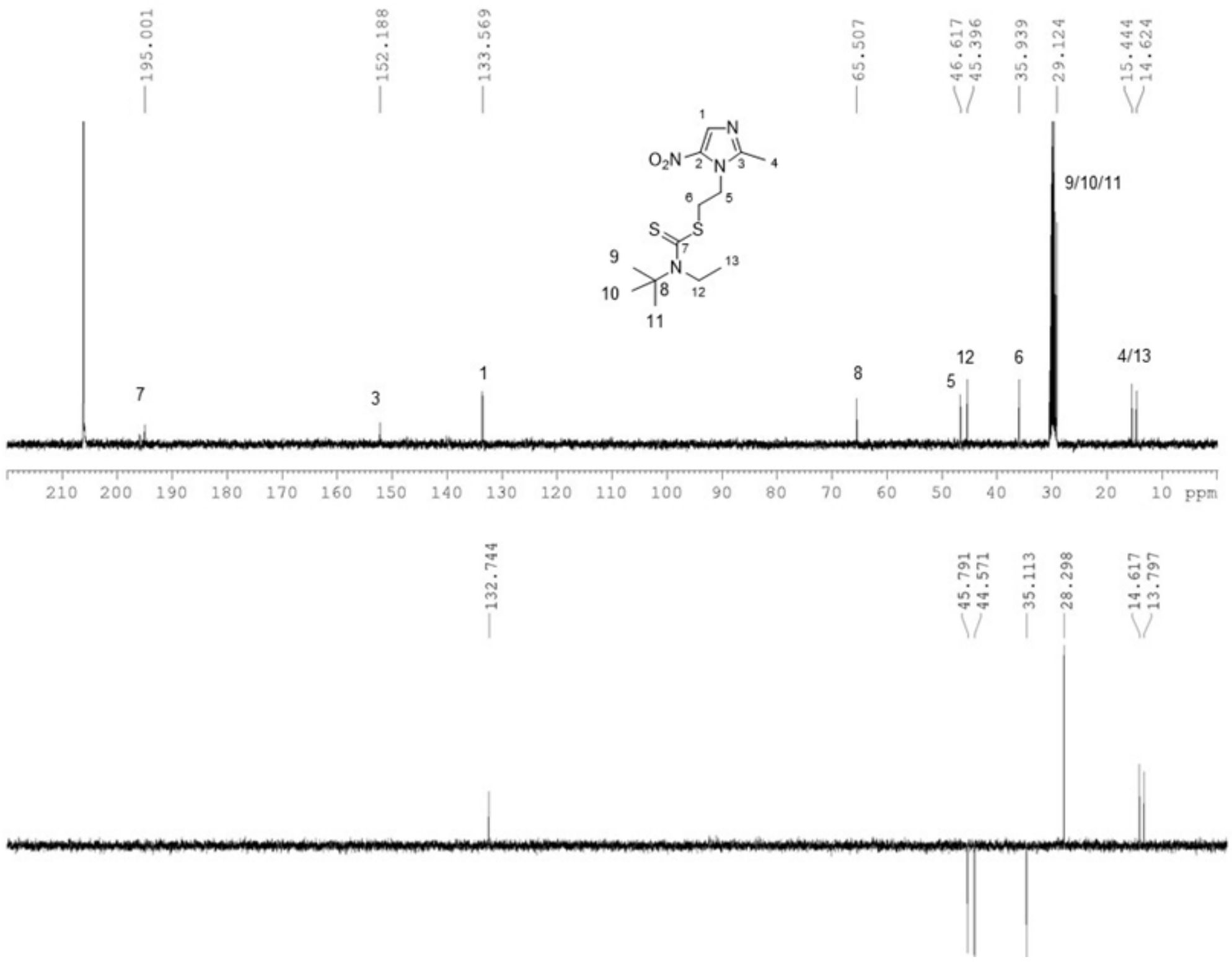

Figure S15. Infrared spectrum of compound 7a

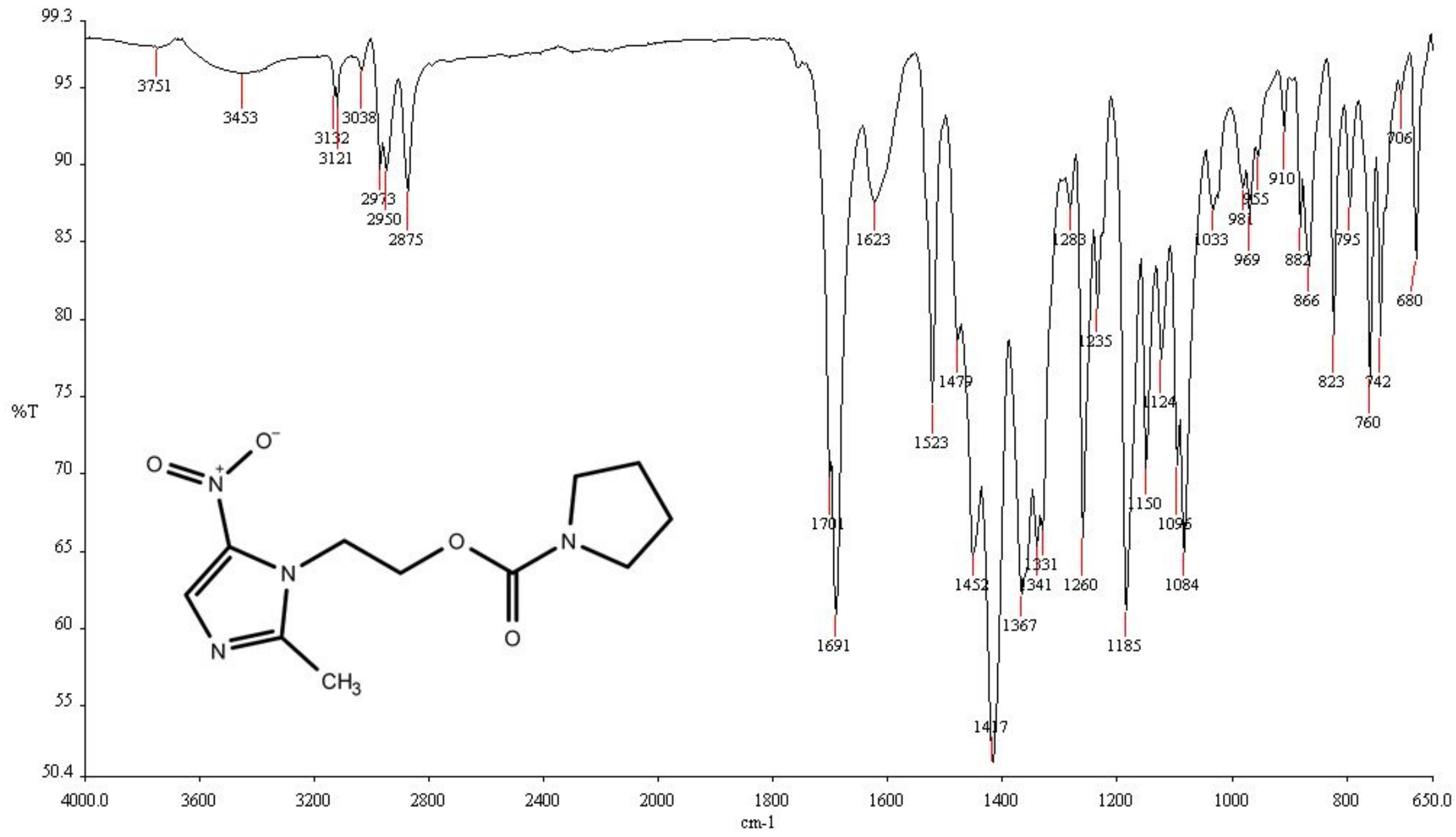

Figure S16.^{1}H NMR (400 MHz, $CDCl_3$) spectrum of compound 7a

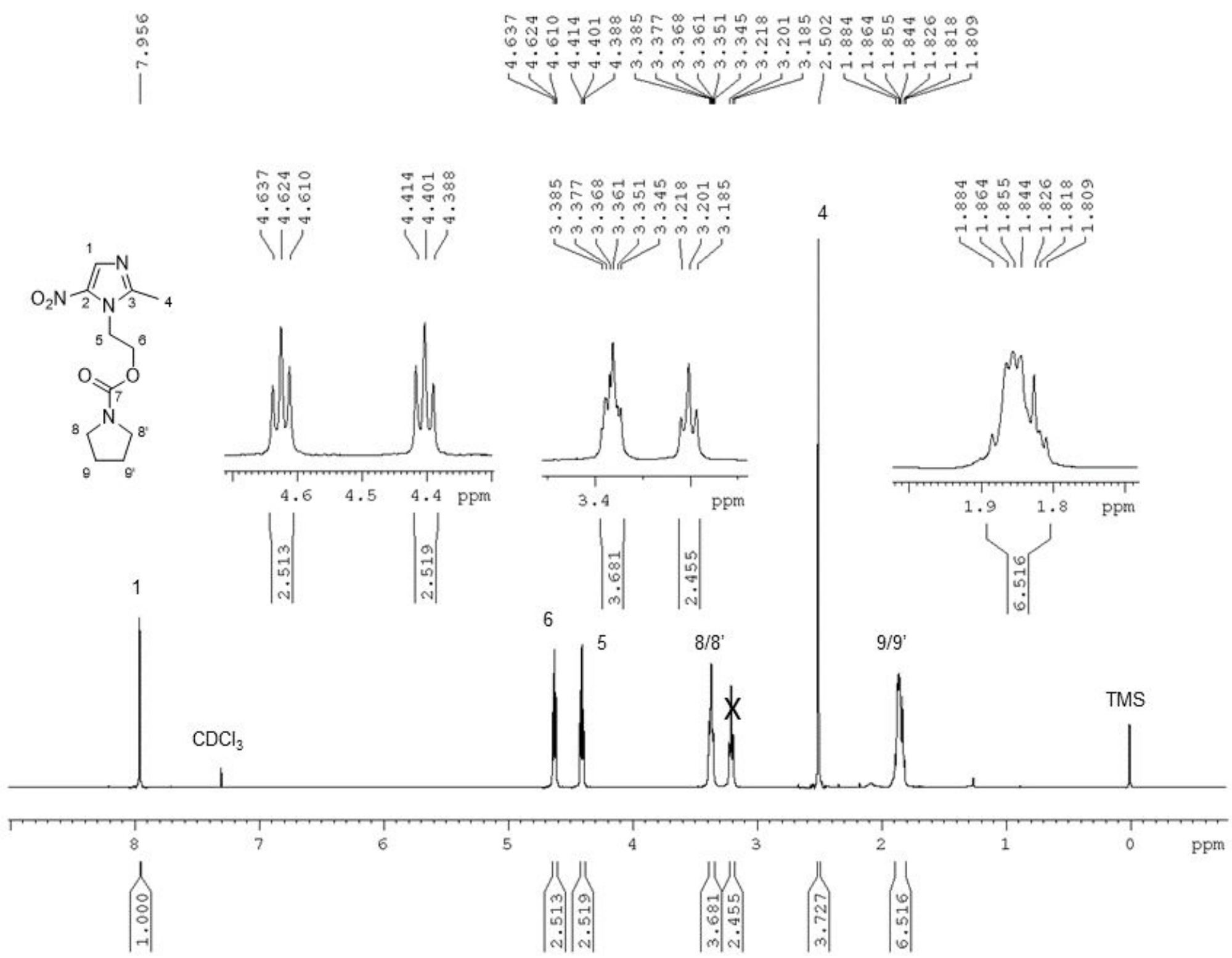

Figure S17.^{13}C and DEPT-135 NMR (100 MHz, $CDCl_3$) spectra of compound 7a

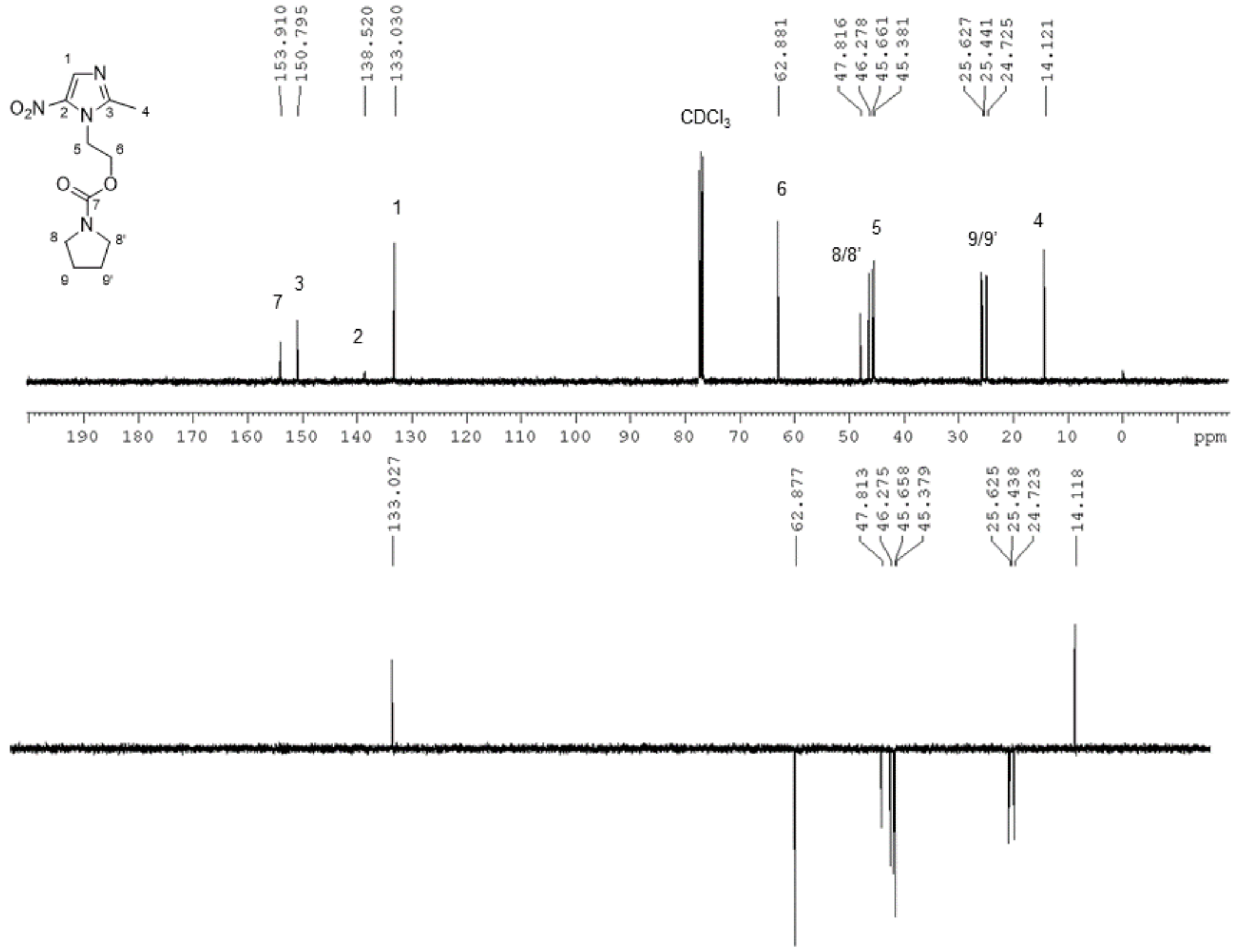

Supplement: Supplementary file 1 [file ao6c05649_si_002.pdf]
